# A Leafhopper-Transmissible DNA Virus with Novel Evolutionary Lineage in the Family *Geminiviridae* Implicated in Grapevine Redleaf Disease by Next-Generation Sequencing

**DOI:** 10.1371/journal.pone.0064194

**Published:** 2013-06-05

**Authors:** Sudarsana Poojari, Olufemi J. Alabi, Viacheslav Y. Fofanov, Rayapati A. Naidu

**Affiliations:** 1 Department of Plant Pathology, Washington State University, Irrigated Agriculture Research and Extension Center, Prosser, Washington, United States of America; 2 Eureka Genomics, Sugar Land, Texas, United States of America; Institute of Infectious Disease and Molecular Medicine, South Africa

## Abstract

A graft-transmissible disease displaying red veins, red blotches and total reddening of leaves in red-berried wine grape (*Vitis vinifera* L.) cultivars was observed in commercial vineyards. Next-generation sequencing technology was used to identify etiological agent(s) associated with this emerging disease, designated as grapevine redleaf disease (GRD). High quality RNA extracted from leaves of grape cultivars Merlot and Cabernet Franc with and without GRD symptoms was used to prepare cDNA libraries. Assembly of highly informative sequence reads generated from Illumina sequencing of cDNA libraries, followed by bioinformatic analyses of sequence contigs resulted in specific identification of taxonomically disparate viruses and viroids in samples with and without GRD symptoms. A single-stranded DNA virus, tentatively named Grapevine redleaf-associated virus (GRLaV), and *Grapevine fanleaf virus* were detected only in grapevines showing GRD symptoms. In contrast, *Grapevine rupestris stem pitting-associated virus*, *Hop stunt viroid*, *Grapevine yellow speckle viroid 1*, *Citrus exocortis viroid* and *Citrus exocortis Yucatan viroid* were present in both symptomatic and non-symptomatic grapevines. GRLaV was transmitted by the Virginia creeper leafhopper (*Erythroneura ziczac* Walsh) from grapevine-to-grapevine under greenhouse conditions. Molecular and phylogenetic analyses indicated that GRLaV, almost identical to recently reported Grapevine Cabernet Franc-associated virus from New York and Grapevine red blotch-associated virus from California, represents an evolutionarily distinct lineage in the family *Geminiviridae* with genome characteristics distinct from other leafhopper-transmitted geminiviruses. GRD significantly reduced fruit yield and affected berry quality parameters demonstrating negative impacts of the disease. Higher quantities of carbohydrates were present in symptomatic leaves suggesting their possible role in the expression of redleaf symptoms.

## Introduction

Nearly seventy viruses and other infectious sub-cellular obligate parasites, collectively referred to as graft-transmissible agents (GTAs), have been documented in grapevines (*Vitis* spp.) [1], [2]. Among all diseases caused either directly or indirectly by these GTAs, grapevine leafroll disease is considered as the most economically important disease affecting plant vigor and longevity and causing significant losses in fruit yield and impacting berry quality attributes [3], [4], [5]. Other virus diseases, such as rugose wood complex, fanleaf infectious degeneration and fleck complex, represent a group of disorders distributed widely in several grape-growing countries around the world [1], [2]. Besides these 'traditional' virus diseases, which can cause significant problems to grape production, other diseases due to GTAs have limited geographic distribution causing relatively less economic damage to grape production.

In addition to viruses, several viroids belonging to the family *Pospiviroidae* are ubiquitous in cultivated grapevines [6], [7], [8], [9]. They are *Hop stunt viroid* (HpSVd, genus *Hostuviroid*), *Grapevine yellow speckle viroid 1* (GYSVd-1, genus *Apscaviroid*) and 2 (GYSVd-2, genus *Apscaviroid*), *Citrus exocortis viroid* (CEVd, genus *Pospiviroid*) and *Australian grapevine viroid* (AGVd, genus *Apscaviroid*). Although these viroids are found in symptomless grapevines, GYSVd-1 has been implicated in vein-banding and yellow speckle symptoms, likely due to a synergistic interaction between GYSVd-1 and *Grapevine fanleaf virus* (GFLV, genus *Nepovirus*, family *Comoviridae*) [10], [11].

Besides their negative impacts on yield and quality of grapes, the introduction and subsequent spread of viruses and other GTAs to healthy vineyards is of great concern for sanitation and grapevine certification programs. Mixed infections of different viruses and viroids are frequent in grapevines and it is believed that such infections can exacerbate symptom severity due to synergistic interactions [12], [13]. Due to clonal propagation of grapevines, viruses can be disseminated via the distribution of planting materials and their introduction into new areas may create serious production constraints because of the ability of viruses to thrive under a variety of circumstances and adapt to diverse and changing ecological niches [14]. As a perennial plant growing in the field for many years, infected grapevines can also serve as constant inoculum sources for secondary spread to other vineyards, if a competent vector is present in the surrounding areas. Additionally, a variety of factors can trigger outbreaks of diseases caused by newly introduced viruses exacerbating crop losses with concomitant negative economic impacts [15]. It is, therefore, imperative that new viruses infecting grapevines are characterized and diagnostic methods established to deal with potential threats they might pose to the viticulture industry in a given grape-growing region.

**Figure 1:**
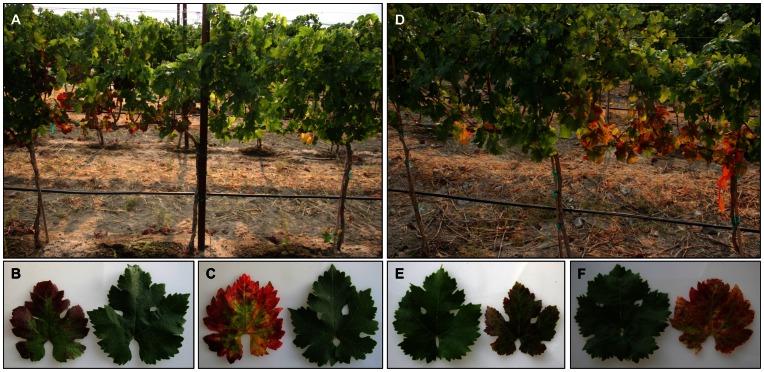
Field symptoms of grapevine redleaf disease in two red-berried wine grape cultivars. (A) Merlot grapevine with symptoms on the left and non-symptomatic grapevine on the right. (B) and (C) Leaves of Merlot showing red veins and irregular blotches in the initial and advanced stages of symptom development, respectively, are on the left and leaves from non-symptomatic grapevines on the right. (D) Cabernet Franc grapevine with symptoms on the right and non-symptomatic grapevine on the left. (E) and (F) Leaves of Cabernet Franc showing discolorations between veins in the early and advanced stages of symptom development, respectively, are on the right and leaves from non-symptomatic grapevines on the left. Symptomatic leaves of GRD-affected grapevines are smaller in size compared to leaves from neighboring non-symptomatic grapevines. Note differences in color of symptomatic leaves between the two cultivars and red veins in Merlot (B–C) and presence of a narrow strip of green tissue on either side of the major veins (green veins) in Cabernet Franc (E–F).

A disease showing red veins, red blotches and total reddening of leaves, designated as grapevine redleaf disease (GRD), has been observed in *V. vinifera* cultivars Merlot and Cabernet Franc (Fig. 1) planted in some commercial vineyard blocks in Washington State, USA. It was not clear whether GRD has been introduced via cuttings imported from outside the state or long existed here but escaped attention in previous years due to symptoms mimicking those produced by the grapevine leafroll disease [3]. The similarity of symptoms to a certain extent with grapevine leafroll disease would suggest that GRD may be caused by infection with grapevine leafroll-associated viruses (GLRaVs) [16]. However, initial diagnostic tests were negative for currently known GLRaVs leading to the hypothesis that a 'new' strain of GLRaV or 'new' virus(es) could be present in grapevines exhibiting GRD symptoms. Since identifying hither-to-unknown viruses in grapevines by traditional virological methods is less efficient and time consuming, next-generation sequencing (NGS) has been employed in recent years for quick identification of viruses and elucidating their possible role in emerging diseases [17], [18].

**Figure 2:**
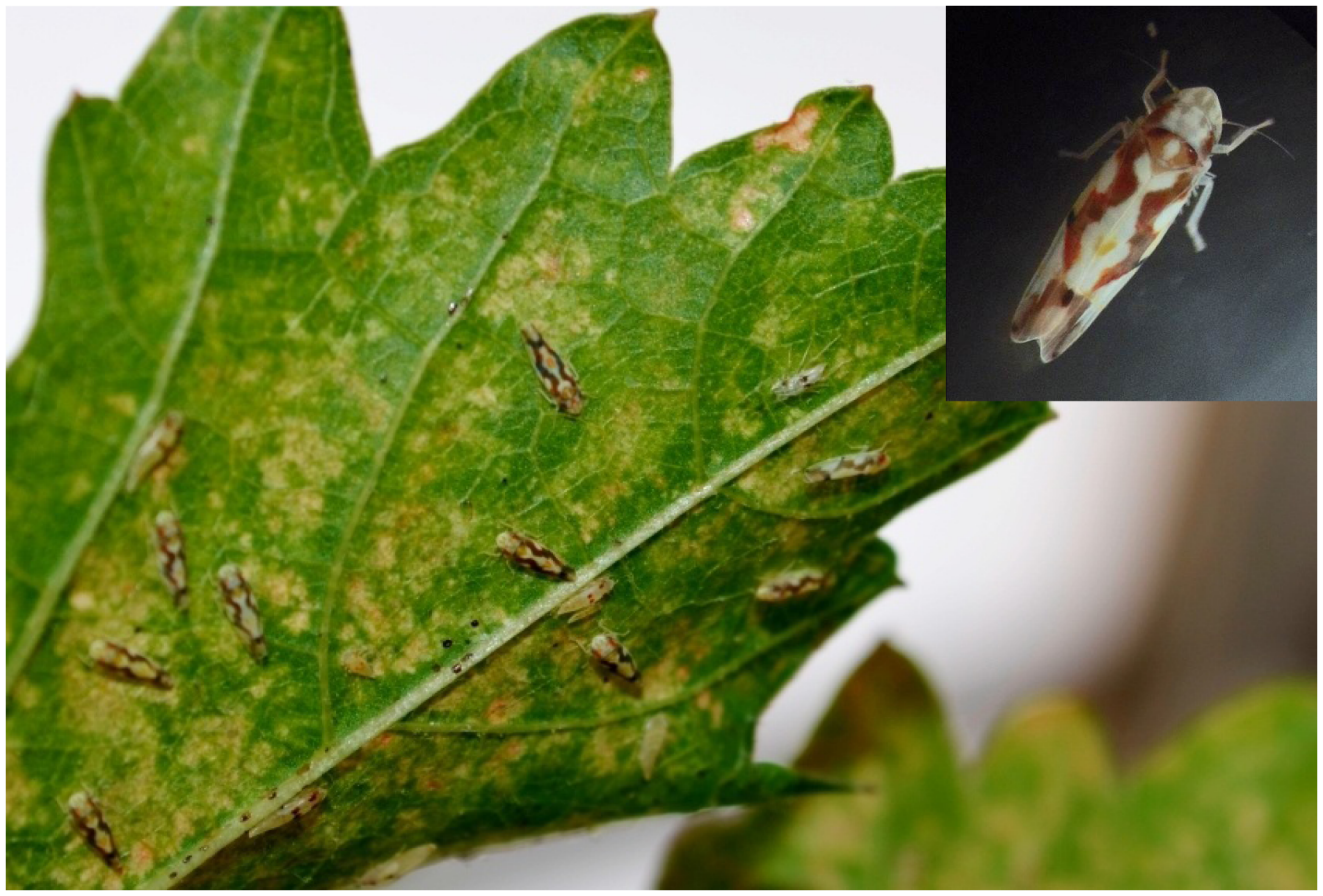
Virginia creeper leafhopper (*Erythroneura ziczac* Walsh) on grapevine leaves. Adult leafhoppers and their feeding damage on the adaxial surface of a grapevine leaf. The inset is a close-up of an adult leafhopper showing reddish-brown eyes, pale yellow to light brownish-yellow body color and a more regular reddish-brown zigzag pattern on the wings.

In this study, Illumina sequencing technology was used to identify taxonomically disparate viruses and viroids in grapevines showing GRD symptoms. One of them is a single-stranded (ss) DNA virus, provisionally designated as Grapevine redleaf-associated virus (GRLaV), which is almost identical to recently described Grapevine Cabernet Franc-associated virus (GCFaV; JQ901105) from New York [19]. Its unique genome organization and phylogenetic relationships indicated that GRLaV represents an evolutionarily distinct lineage in the family *Geminiviridae*. Our study revealed that GRLaV is transmitted by the Virginia creeper leafhopper (*Erythroneura ziczac* Walsh; Hemiptera: Cicadellidae; Fig. 2) commonly found as a pest on grapevines [20]. To the best of our knowledge, this is the first report of the transmission of a grapevine-infecting geminivirus by a leafhopper species in the genus *Erythroneura*. Our results also showed that GRD significantly affects grapevine performance, fruit yield and berry quality attributes, demonstrating its negative impacts on wine grape cultivars.

## Materials and Methods

### Ethics Statement

Specific approval was obtained from the owner of a commercial vineyard to collect samples. Name of the location and owner of this private property is withheld due to confidentiality agreement with the grower. Data on yield and berry quality analyses were collected from the same vineyard with permission from the grower. This study did not involve endangered or protected species.

### Plant Material

Leaf samples were collected from six year-old, own-rooted Merlot and Cabernet Franc wine grape cultivars. These grapevines were planted in a commercial vineyard located near Prosser in Washington State, USA (46.2°N latitude, 119.8°W longitude). The grapevines were grown under standard viticultural practices with drip irrigation. Leaves with symptoms from GRD-affected grapevines and comparable leaves with no symptoms from neighboring non-symptomatic grapevines (Fig. 1A and 1D) were collected in September 2011 and 2012. The leaf samples were frozen in liquid N_2_ immediately after harvesting and stored subsequently at −80°C until further analysis.

### Extraction of Total RNA and Quality Control

Total RNA was extracted separately from leaves of four symptomatic and four non-symptomatic Merlot and Cabernet Franc grapevines using Spectrum Plant Total RNA kit (Sigma-Aldrich, St Louis, MO, USA) following the manufacturer’s instructions. After treating with DNase I (Qiagen Inc., Valencia, CA, USA), RNA concentration was estimated using a Nanodrop ND-1000 spectrophotometer (NanoDrop Technologies, Rockland, DE, USA). Total RNA with an A_260_/A_280_ ratio above 1.8 was used to determine RNA integrity number (RIN) by the Agilent 2100 Bioanalyzer (Agilent Technologies, Palo Alto, CA, USA). Only RNA with RIN ≥8.0 was used for subsequent studies. Equal quantities of RNA from Merlot and Cabernet Franc grapevines were pooled such that one batch of high quality total RNA represented symptomatic leaves of GRD-affected grapevines and another represented leaves with no symptoms from neighboring non-symptomatic grapevines.

### Pre-amplification Enrichment of Target RNA and Preparation of cDNA Libraries

Five micrograms of total RNA each for symptomatic and non-symptomatic samples was subjected separately to removal of ribosomal (r)RNA using Epibio’s Ribo-Zero rRNA Removal Kit for Plant Leaf (Epicenter, Madison, WI, USA) according to the manufacturer’s instructions. The rRNA-depleted (ribo-depleted) RNA samples were fragmented, followed by first-strand cDNA synthesis using random hexamers and Superscript II (Life Technologies, Grand Island, NY, USA). After second-strand synthesis, the cDNAs were end-repaired, A-tagged, ligated to Illumina’s paired-end index adaptor, size-selected from an agarose gel, and enriched with 18 cycles of PCR. Separately, 15 µg of total RNA each from symptomatic and non-symptomatic leaves was similarly ribo-depleted as described above. Ribo-depletion was followed by poly-A selection to remove mRNA from the samples. The resulting ribo- and mRNA-depleted samples (dual-depleted samples) were fragmented and used for preparing cDNA libraries using Illumina’s mRNA-seq Sample Prep kit (Illumina Inc., San Diego, CA, USA) according to the manufacturer’s instructions.

### Next-generation Sequencing and Analysis of cDNA Libraries

The cDNA libraries prepared from ribo-depleted and dual-depleted RNA samples were subjected to NGS using the Sequencing-By-Synthesis Technology (Illumina Inc., San Diego, CA, USA) on the Illumina Genome Analyzer IIx (www.eurekagenomics.com). The deep-sequence data set obtained from NGS was deposited in the NIH Short Read Archive as accession numbers SRX245253 for the PE 51 mers and SRX245255 for the SE 36 mers. Initially, sequence reads from each cDNA library were mapped to the reference genome of *V. vinifera* (PRJEA18785). Reads not mapped to *V. vinifera* were used for further analysis. An alignment-based approach was followed to identify virus- and viroid-specific sequence reads in symptomatic and non-symptomatic samples. For this approach, a non-redundant virus/viroid database was created representing 51,696 sequence records of viruses and viroids infecting plants and fungi. The sequence reads were mapped to the virus/viroid database using mapping software BWA version 0.6.2 [21] tolerating up to 3 mismatches. Candidate virus/viroid sources were chosen on the basis of the number of hits from sequence reads obtained from symptomatic and non-symptomatic samples (Table 1).

**Table 1:** Classification of pathogen-specific sequence reads from symptomatic and non-symptomatic samples.

Category of sequence reads	Reads from ribo-depletedcDNA library		Reads from dual-depletedcDNA library	
	Symptoms	No Symptoms	Symptoms	No Symptoms
**Reads mapped to specific virus**				
GRLaV	15,036	0	406	0
GFLV	14,383	0	978	0
GRSPaV	662	1,353	42	68
**Reads mapped to specific viroid**				
GYSVd-1	13,914	9,004	987	490
CEYVd	1,605	152	5	10
CEVd	856	86	6	2
HpSVd	1,964	2,980	164	185
Other*	5,568	2,693	644	1,604
**Total**	53,988	16,268	3,232	2,539

GRLaV = Grapevine redleaf-associated virus, GRSPaV = Grapevine rupestris stem pitting-associated virus, GFLV = Grapevine fanleaf virus, HpSVd = Hop stunt viroid, GYSVd-1 = Grapevine yellow speckle viroid 1, CEVd = Citrus exocortis viroid, CEYVd = Citrus exocortis Yucatan viroid.

* Reads mismapped to virus- or viroid-like sequences.

Classification and abundance of high-throughput sequence reads obtained from ribo-depleted and dual-depleted cDNA libraries were determined by mapping the reads onto the virus/viroid database using mapping software BWA 0.6.

Multiple alignments of candidate virus/viroid nucleotide (nt) and amino acid (aa) sequences were performed using ClustalX [22] and MUSCLE [23] respectively, with default parameters. Percentage sequence identities were computed using Vector NTI Advance 11 software (Life Technologies, Grand Island, NY, USA). Neighbor-joining (NJ) and Maximum likelihood (ML) phylogenetic trees were constructed using the MEGA 5 software package [24] with 1,000 bootstrap replicates and branches with less than 70% bootstrap values were collapsed.

### Detection of Viruses and Viroids in Grapevine Samples

Virus- and viroid-specific sequences generated from the NGS data were used to design primers using Primer Express 3.0 (Life Technologies, Grand Island, NY, USA). Total RNA extracted from petiole samples collected from individual source grapevines, originally used for NGS, was used as template in one step-single tube RT-PCR for the detection of GFLV, *Grapevine rupestris stem pitting-associated virus* (GRSPaV), HpSVd, GSYVd-1, CEVd, and *Citrus exocortis Yucatan viroid* (CEYVd) using species-specific primers (Table 2) and conditions described earlier [25], [26], [27]. For the detection of GRLaV, DNA was isolated [28] from leaf tissue collected from the same grapevines and used as a template in PCR assays. The primer pair GRLaV-For and GRLaV-Rev (Table 2) was used to amplify a 557 base pair (bp) DNA fragment encoding partial intergenic region (IR) and coat protein (CP) region of GRLaV. PCR conditions were an initial denaturation at 95°C for 3 min followed by 35 consecutive cycles of 95°C for 30s, 57°C for 45s and 72°C for 30s, and a final extension of 72°C for 10min in a GeneAmp PCR System 9700 (Life Technologies, Grand Island, NY, USA). Additional primers (Table 2) were designed to amplify the complete genome of GRLaV as four overlapping DNA fragments. In all cases, amplicons were cloned into pCR2.1-TOPO vector (Life Technologies, Grand Island, NY, USA) and three independent clones per amplicon were sequenced in both orientations. Nucleotide sequences were analyzed and pairwise comparisons were made with corresponding NGS-derived sequences and sequences available in GenBank using Vector NTI Advance 11 (Life Technologies, Grand Island, NY, USA).

**Table 2:** Oligonucleotide primers used for amplification of virus- and viroid-specific sequences by RT-PCR and PCR.

Name	Sequence	Genome Position (from 5′end)
**For genome characterization of GRLaV:**		
GRLaV-1-For	CCCATGGTACGTGGTATTCTTGCG	11–34
GRLaV-1-Rev	CAGTTCCAGTAGGAAACCGATC	1033–1054
GRLaV-2-For	GGCCTCGTAGTAGGCCTTGTC	811–831
GRLaV-2-Rev	GCAACATTCAAGCCGTGGGCTG	1967–1988
GRLaV-3-For	GCATAGTCCAGACAGTCGTTGTAC	1728–1751
GRLaV-3-Rev	GCCCAGAGATGTCGCCGACGTGC	2778–2800
GRLaV-4-For	GTAGATTGAGGACGTATTGG	2601–2620
GRLaV-4-Rev	CGCAAGAATACCACGTACCATGGG	34–11
**For detection of viruses and viroids by PCR/RT-PCR:**		
GRLaV-For	CTCGTCGCATTTGTAAGA	255–272
GRLaV-Rev	ACTGACAAGGCCTACTACG	793–811
GFLV-For	ACTGGTTTGACGTGGGTGAT	2224–2243 (RNA-2)
GFLV-Rev	CCAAAGTTGGTTTCCCAAGA	2526–2545 (RNA-2)
GRSPaV-For	GATGAGGTTCAGTTGTTTC	4372–4390
GRSPaV-Rev	TCACCAAATGTGAGAGTGAGCTG	4771–4793
HpSVd-For	GAGCCCCGGGGCAACTCTTCTC	74–95
HpSVd-Rev	TTTCTCAGGTAAGTACCTCCCTG	50–72
GYSVd-1-For	TGCCTCCGCTAGTCGAGCGG	254–273
GYSVd-1-Rev	CGACGACGAGGCTCACT	88–104
CEVd/CEYVd-For	GGAAACCTGGAGGAAGGTG	9–27
CEVd/CEYVd Rev	CCGGGTACATATTCACCGCGGCA	206–228

GRLaV = Grapevine redleaf-associated virus, GRSPaV = Grapevine rupestris stem pitting-associated virus, GFLV = Grapevine fanleaf virus, HpSVd = Hop stunt viroid, GYSVd-1 = Grapevine yellow speckle viroid 1, GYSVd-2 = Grapevine yellow speckle viroid 2, CEVd = Citrus exocortis viroid, CEYVd = Citrus exocortis Yucatan viroid, For = forward (sense) primer, Rev = reverse (antisense) primer.

### Transmission of GRD and Associated Viruses and Viroids

#### Transmission by grafting

Single-budded dormant cuttings of virus-free Cabernet Franc obtained from a Certified Nursery were used as scion to bench ( = Omega) graft onto rootstock cuttings of approximately the same diameter collected from dormant symptomatic and non-symptomatic Merlot and Cabernet Franc grapevines. After adequate callus development around the graft union, grafted cuttings were planted individually in 4-inch pots and maintained in the greenhouse for initial establishment and subsequently planted in an experimental vineyard in the spring of 2011. Fifteen grafted plants each for symptomatic and non-symptomatic grapevines per cultivar were planted at a spacing of 1.8 m within rows and 3 m between rows. Grapevines were trained to a single trunk with bilateral cordons at 1.2 m above the ground, drip-irrigated and managed by controlling weeds and pests according to standard viticultural practices. Buds and shoots emerging from rootstocks were clipped regularly to promote the emergence of scion buds. Leaves on growing scion shoots were observed at regular intervals for symptom development between June and October during the 2012 season. Leaf samples were harvested in October from grafted grapevines and tested by RT-PCR and PCR described above for confirmation of viruses and viroids identified by NGS.

#### Vector transmission

Cuttings from symptomatic Merlot and Cabernet Franc grapevines, collected from source grapevines used for NGS, were planted in 12-inch pots as own-rooted plants and maintained in insect-proof cages in the greenhouse at 25±2°C with 14 hr daylight. Cohorts of about 50 adult Virginia creeper leafhoppers (Fig. 2), reared on virus-free grapevines, were allowed to feed on leaves of GRLaV-positive plants for a 72-hr acquisition access period (AAP). Twenty to twenty five potentially viruliferous leafhoppers were collected and released on 3-month old virus-free grapevines, raised from seeds of Cabernet Franc, Merlot, Chardonnay and Pinot Noir and cuttings of Pixie (a dwarf Pinot Meunier), and maintained in insect-proof cages. After a 72-hr inoculation access period (IAP), leafhoppers were killed by applying Assail 30 SG at 7.5 g/gallon. Five inoculated grapevines per cultivar were subsequently maintained in insect-proof cages in the greenhouse. Corresponding non-inoculated grapevines were maintained in separate cages to serve as controls. Newly emerging leaves from inoculated and non-inoculated grapevines were sampled four weeks post-IAP and tested by PCR for the presence of GRLaV as described above.

### Impacts of GRD on Grapevine Performance and Fruit Maturity Indices

To minimize errors in experimental results due to variations in growing conditions, symptomatic and non-symptomatic Merlot and Cabernet Franc grapevines were selected in the commercial vineyard blocks such that individual grapevines exhibiting GRD symptoms were adjacent to non-symptomatic grapevines in the same row. Eight pairs of symptomatic and non-symptomatic grapevines distributed in four rows in each block of Merlot and Cabernet Franc were used for this study conducted during 2011 and 2012 seasons. At the beginning of the growing season, the length of growing shoots from individual grapevines was measured using a flexible tape at two time points, four weeks apart, prior to 50% bloom. At commercial harvest, the number of clusters produced by individual grapevines was counted and their combined weight quantified using a digital SVI-50C weighing scale (Acculab, Edgewood, NY). During the winter of 2012, dormant canes were pruned from individual grapevines and their combined weight quantified using a digital SVI-50C weighing scale.

For fruit composition analyses, 20 berries were collected randomly at the time of commercial harvest from clusters of individual symptomatic and non-symptomatic grapevines and stored in sealed Ziploc bags under cool conditions while in transit to the laboratory. Berries from symptomatic and non-symptomatic grapevines were pooled separately, divided randomly into five replicates of 30 berries each, and homogenized in an analytical grinding mill (A11, IKA® Works, Inc., Wilmington, NC). The homogenate was clarified by low speed centrifugation at ∼2,000 g and the supernatant used for measuring fruit maturity indices; namely, total soluble solids (TSS), titratable acidity (TA) and pH. TSS concentration (Brix) was measured using a PAL-1 Digital Pocket Refractometer (Atago Co. Ltd., Tokyo, Japan). TA was determined by direct titration with 0.1 N NaOH to an endpoint of pH 8.2 using a Mettler-Toledo DL50 Rondolino Autotitrator (Mettler Toledo Inc., Columbus, OH) and expressed as tartaric acid equivalents. Juice pH was measured with a MP225 pH meter (Mettler Toledo Inc., Columbus, OH). Total anthocyanins were extracted from berry samples and their quantity measured according to Iland *et al*. [29]. In addition, four pairs of mature leaves at the 3^rd^ and 4^th^ nodes from the basal part of primary canes of symptomatic and non-symptomatic Merlot and Cabernet Franc grapevines were harvested during the fourth week of September 2011 and processed for quantifying soluble carbohydrates (glucose, sucrose, fructose and starch) as described by Zhao *et al.* [30]. All data were subjected to factorial analysis of variance (ANOVA) using the SAS statistical package (SAS Institute, Cary, NC) to determine significant differences between symptomatic and non-symptomatic grapevines.

## Results

### GRD Symptoms in Cultivars Merlot and Cabernet Franc

No foliar symptoms were observed in Merlot and Cabernet Franc grapevines before *véraison* (onset of berry ripening). Soon after *véraison*, which begins normally in the first half of August under Washington conditions, inter-veinal areas of mature leaves at bottom portions of primary canes started showing small, irregular red-colored areas in both cultivars. As the season progressed, these discolored areas expanded, coalesced forming irregular blotches and became more apparent by the end of the season in Merlot (Fig. 1A–C). In some leaves, these irregular blotches were restricted to leaf margins. Overall, symptoms in Merlot ranged from red veins, red blotches, and total reddening of the leaves. In the case of Cabernet Franc, mature leaves exhibited mild reddening of inter-veinal portions of the lamina with a narrow strip of tissue on either side of the major veins largely remaining green (Fig. 1D–F). In some leaves, discontinuous red blotches were restricted to leaf margins. In general, symptomatic leaves of Merlot exhibited deep-red color and those of Cabernet Franc showed purple-red color. In both cultivars, symptomatic leaves did not show downward rolling of margins, a characteristic symptom of grapevine leafroll disease [3]. Also, symptoms in both cultivars were confined to mature leaves at bottom portions of the canopy with leaves at top portions remaining non-symptomatic (Fig. 1A and 1D).

### NGS Reveals the Presence of Disparate Viruses and Viroids in Grapevines Showing GRD Symptoms

A total of 34,504,862 and 76,668,556 sequence reads were obtained from ribo-depleted cDNA libraries derived from symptomatic and non-symptomatic samples, respectively. Among these reads, a total of 22,566,023 and 42,209,161 reads from symptomatic and non-symptomatic samples, respectively, that did not map to *V. vinifera* genome were used to map against the non-redundant virus/viroid database (Table 1 and Table S1). Out of these reads, a total of 53,988 and 16,268 hits (Table 1) from symptomatic and non-symptomatic samples, respectively, aligned with sequences in the virus- and viroid-specific sequence database. Overall, the data showed the presence of sequences specific to GRLaV and GFLV only in reads generated from symptomatic leaves. Sequences specific to GRSPaV, HpSVd, GYSVd-1, CEVd and CEYVd were present in reads generated from both symptomatic and non-symptomatic samples. Contigs built from these sequences allowed the generation of complete genome coverage for GRLaV (KC427993-96), GRSPaV (KC427107), GYSVd-1 (KC427099-102), HpSVd (KC427095-98), CEYVd (KC427105-106) and CEVd (KC427103-04), and near complete coverage for GFLV RNA-1 (92%) and RNA-2 (86%) (KC427991-92) (Fig. S1). The majority of hits to the virus/viroid database in sequence reads generated from symptomatic leaves were specific to GRLaV followed by GFLV, accounting for a higher number of total hits compared to non-symptomatic leaves (Table 1).

In the case of dual-depleted samples, a significantly lower number of total sequence reads were obtained when compared to corresponding reads from ribo-depleted samples (Table 1 and Table S1). As a result, fewer hits were aligned with the virus- and viroid-specific sequence database resulting in incomplete coverage of virus and viroid genomes identified above. However, the results correlated well with ribo-depleted data (Table 1) and similar conclusions were reached with both approaches.

### Molecular Analysis of the GRLaV Genome Shows Distinct Differences with Members of the Family *Geminiviridae*

**Figure 3:**
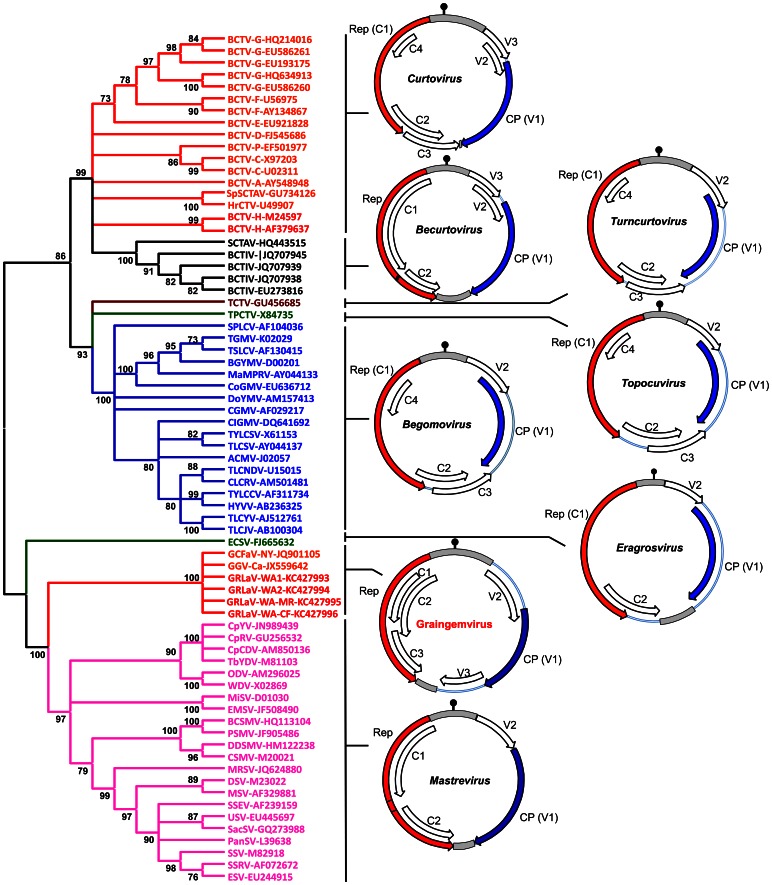
Phylogenetic analysis of Grapevine redleaf-associated virus (GRLaV). The neighbor-joining phylogenetic tree was constructed using full-length genomes of GRLaV and members of approved genera in the family *Geminiviridae*. The consensus tree on the left was drawn to scale with bootstrap values (1,000 replicates) shown at the nodes. Branches with less than 70% bootstrap support have been collapsed. The genome organization of GRLaV and representative members of approved genera in the family *Geminiviridae* is shown on the right. Position and orientation of individual ORFs is indicated by arrows with clockwise direction representing virion sense and anticlockwise direction representing complementary sense. The coat protein (CP) and replication-associated protein (Rep) are shown in blue and red color, respectively. Intergenic regions are represented as grey boxes. The location of the putative stem-loop (hairpin) structure at the origin of virion strand replication within the large intergenic region is indicated in black at the 12 O’clock position. Genome maps were drawn using guidelines described in ICTV taxonomy proposal 2012.018a-p to P2012.018pP.

The genome of GRLaV (KC427993-96) was determined to be 3,208 nt in size and encodes three overlapping open reading frames (ORFs), designated as V1, V2 and V3, on the spliced virion strand and three ORFs (C1, C2 and C3) on the spliced complementary sense orientation of the genome (Fig. 3). GRLaV showed 99.6% identity with GCFaV (JQ901105) from New York [19] and 99% identity with a geminivirus isolate from British Columbia (JX559642). However, GRLaV differed from these two isolates in having an extra two nucleotides at positions 14 and 1841, respectively. Sequencing of several independent clones confirmed that the presence of two additional nucleotides in GRLaV genome was not due to misincorporation during PCR amplification or errors introduced during sequencing or sequence assembly. The two extra nucleotides were located in non-coding regions of the genome between the origin of replication and ORF V2 (position 14) and between ORFs V3 and C3 (position 1841) in the genome with no effect on the integrity of ORFs encoded by the virus. Pairwise comparison of multiple alignments of individual ORFs indicated that ORFs encoded by GRLaV shared 99–100% and 98–100% identities at the nt and aa levels, respectively, with corresponding ORFs of virus isolates from New York [19] and British Columbia (JX559642). Based on these results, it can be concluded that all three ssDNA virus isolates from grapevines represent sequence variants of the same virus.

In addition to four established genera (*Mastrevirus*, *Curtovirus*, *Topocuvirus* and *Begomovirus*), three additional genera (*Becurtovirus*, *Turncurtovirus* and *Eragrosvirus*) have been approved in the family *Geminiviridae* by the Executive Committee of the International Committee on Taxonomy of Viruses (ICTV) and are currently awaiting ratification (ICTV taxonomy proposal 2012.018a-p to P2012.018pP). A comparison of the genome organization of GRLaV with genomes of type members of these genera (Fig. 3) showed similarities as well as distinct differences, especially in the location of certain ORFs in the virus genome. Similar to becurto-, eragros-, and mastreviruses, GRLV has large (LIR) and small (SIR) intergenic regions separating the virion and complementary sense ORFs. Like most geminiviruses, the LIR contains the TAATATT↓AC nonanucleotide at the viral origin of replication. The V1 and C1 ORFs are positional and functional analogues, respectively, of the CP and replication-initiator or replication-associated (Rep) genes of well characterized geminiviruses. Similar to curto- and becurtoviruses, GRLaV encodes three ORFs in the virion sense. However, the location of these ORFs differ in GRLaV in that both V2 and V3 ORFs are located upstream of the CP in curto- and becurtoviruses whereas the V3 ORF of GRLaV is located downstream of the CP (Fig. 3), thus making GRLaV unique among the currently recognized members of the family *Geminiviridae*.To further elucidate relationships at the molecular level between GRLaV and currently recognized geminiviruses, we performed multiple alignments using amino acid sequences of Rep gene (Fig. S2) and scanned for the presence of conserved motifs at the N-terminus as described by Nash *et al*. [31]. The dsDNA binding motif I of GRLaV contains amino acid residues ‘FLTYP’ and shares 100% conservation with viruses belonging to the genera *Curtovirus*, *Turncurtovirus* and *Topocuvirus*. In contrast, it shares 80–100% conservation with members belonging to the genera *Becurtovirus*, *Begomovirus*, *Mastrevirus* and *Eragrosvirus*. The metal-binding motif II of GRLaV contains residues ‘HLHALL’ that shows 33% conservation among currently described geminiviruses. Interestingly, motif II of GRLaV is 100% identical only to that of *Maize streak virus*, a monopartite leafhopper-transmitted ‘Old World’ virus. Motif III of GRLaV contains amino acid residues ‘YVGKE’ and pairwise alignment of this catalytic site for DNA cleavage showed 40% conservation with currently described geminiviruses. The GRS motif of GRLaV is most similar to members of the genus *Mastrevirus*, sharing maximum conservation of 43% with GRS motifs of three monocot-infecting mastreviruses, *Paspalum striate mosaic virus*, *Miscanthus streak virus* and *Maize streak Reunion virus* (Fig. S2). These results indicated that the Rep gene of GRLaV shares several molecular signatures with curtoviruses and mastreviruses than with other geminiviruses.

### Phylogenetic Analysis Suggests that GRLaV Represents a Highly Divergent Lineage within the Family *Geminiviridae*

**Figure 4:**
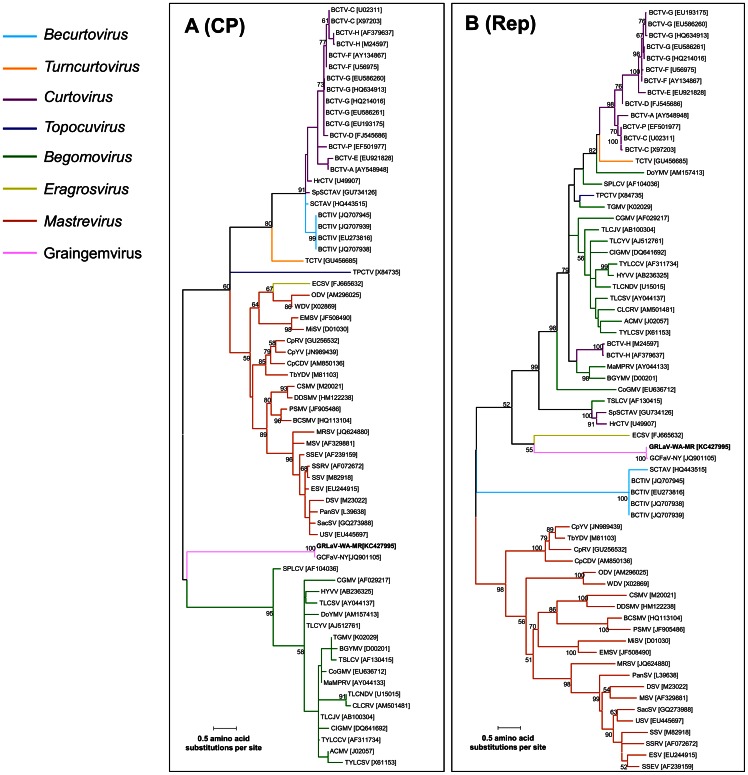
Phylogenetic analysis of Grapevine redleaf-associated virus (GRLaV) using (A) coat protein and (B) replication-associated protein. Maximum likelihood trees were constructed using amino acid sequences of the coat protein (CP) and replication-associated protein (Rep) of GRLaV and representative members of approved genera in the family *Geminiviridae*. The consensus tree for each gene was drawn to scale using the MEGA5 with the rtREV amino acid substitution model and bootstrap values (1,000 replicates) shown at the nodes. Branches with less than 70% bootstrap support have been collapsed. Colored lines in both phylograms correspond to respective genera of the *Geminiviridae* family listed on the top left corner.

In order to determine the phylogenetic relationship of GRLaV with members of the family *Geminiviridae*, neighbor-joining phylogenetic analysis was carried out using the complete genome of GRLaV and representative members of the genera *Begomovirus*, *Curtovirus*, *Becurtovirus*, *Turncurtovirus*, *Topocuvirus*, *Eragrosvirus* and *Mastrevirus*. As shown in Fig. 3, GRLaV formed a distinct clade sandwiched between the genera *Eragrosvirus* and *Mastrevirus*. To decipher possible evolutionary history for GRLaV, maximum-likelihood phylogenetic analyses was carried out using amino acid sequences of the CP and Rep genes, since both genes are known to be relatively well conserved among geminiviruses and are used for taxonomic considerations. For constructing the Rep-based phylogenetic tree, full-length Rep protein expressed from unspliced complementary strand transcript (Fig. 3) in topocuviruses, eragrosviruses, begomoviruses, curtoviruses and GRLaV was used. Since the Rep protein is expressed from a spliced complementary strand transcript in mastreviruses and bacutoviruses, the intergenic region was deleted and the amino acid sequence of RepA and RepB proteins joined end-to-end was used for phylogenetic analysis. In both CP- and Rep-based phylogenetic trees (Fig. 4A and 4B), GRLaV formed a distinct clade, further indicating that it is distinct from currently described members of the family *Geminiviridae*. In the CP-based phylogram (Fig. 4A), the GRLaV clade was positioned between the clades formed by *Begomovirus* and *Mastrevirus*. In the phylogram based on full-length Rep protein (Fig. 4B), the GRLaV clade was located between clades formed by *Becurtovirus* and *Eragrosvirus*. Based on these results, it can be concluded that GRLaV represents an evolutionarily distinct and genus-level member of the family *Geminiviridae*.

### Sequence Analyses of Other Viruses and Viroids Recovered by NGS

Pairwise comparison of GFLV RNA-1 and RNA-2 genome sequences showed 95–99% nt identity with corresponding sequences of GFLV isolates previously reported from Merlot (GQ332370) and Cabernet Franc (GQ332371-72) in Washington vineyards [26]. The 8,742 nt genome of GRSPaV isolates recovered from both symptomatic and non-symptomatic samples showed 100% nt sequence identity with each other and 91–98% nt identity with corresponding sequences of GRSPaV isolates (FJ943344, FJ943333, FJ943295) previously reported from Washington vineyards, and all of these sequences were aligned with the GRSPaV-SG1 lineage [32]. The 366 nt genome sequences of GYSVd-1 obtained in this study (KC427099-102) were 99–100% identical to corresponding sequence of other GYSVd-1 isolates (AB028465, EU682452 and DQ371466) from Washington [27] and other grapevine-growing regions. The 296 nt HpSVd genome sequences obtained in this study (KC427095-98) showed 100% identity with an isolate of HpSVd (GU327606) from Washington [27]. The complete genome sequences of CEVd (KC427103-104) and CEYVd (KC427105-106) are 394 and 395 nts in size, respectively. Both viroids shared 100% identity with corresponding sequences of CEVd (DQ318790) and CEYVd (FJ751934) available in GenBank. To our knowledge, this is the first report of the occurrence of CEYVd in grapevines anywhere in the world and the first report of CEVd in grapevines in the USA.

### GRD is Graft Transmissible

In order to verify if symptoms of GRD can be reproduced by graft inoculation, single-budded cuttings of virus-free Cabernet Franc, a sensitive cultivar used as biological indicator for graft indexing of grapevine leafroll disease, were grafted onto rootstock cuttings derived from Merlot and Cabernet Franc grapevines showing GRD symptoms. The grafted grapevines, planted in the field during the spring of 2011, were observed for GRD symptoms between June and October of 2012. Soon after *véraison*, inter-veinal areas of mature leaves started showing small, irregular red-colored areas in Cabernet Franc scions grafted on rootstocks derived from symptomatic grapevines of Merlot and Cabernet Franc. As the season progressed, the discolored areas expanded, coalesced and became more apparent by the end of the season (Table 3). These symptoms were similar to those observed in source grapevines (Fig. 1). No symptoms were observed before *véraison* and no symptoms were evident on Cabernet Franc scions grafted on rootstocks derived from non-symptomatic grapevines of Merlot and Cabernet Franc. These results indicated that the pattern of development of GRD symptoms in grafted grapevines is similar with those observed in own-rooted source grapevines in the commercial vineyard. It also shows that GRD is graft transmissible with reproducible symptoms. To confirm the presence of viruses and viroids in grafted grapevines showing GRD symptoms, samples were tested by PCR and RT-PCR using species-specific primers (Table 2). The results (Table 3) showed presence of GRLaV in symptomatic scion materials grafted onto GRD-affected Merlot and Cabernet Franc rootstocks, and GRSPaV and HpSVd in scion materials grafted onto GRD-affected and control grapevines of both cultivars. GFLV was detected only in 10% (3/30) of scion materials grafted onto GRD-affected Merlot grapevines and GYSVd-1 in a few scion materials grafted onto GRD-affected and control grapevines of both cultivars (Table 3). Both CEVd and CEYVd were not detected in scion materials grafted onto GRD-affected and control grapevines of both cultivars (Table 3). A possible explanation for the observed differences could be that GFLV, GYSVd-1, CEVd and CEYVd were present at the time of sampling in quantities undetectable by PCR-based diagnostic assay. It could also be that the complex interactions between disparate viruses and viroids in the rootstock favor a more efficient graft transmission of GRLaV, GRSPaV and HpSVd over GFLV and the other three viroid species.

**Table 3:** Graft transmission of grapevine redleaf disease (GRD) and associated viruses and viroids.

Rootstock†	Scion*	Scion materials positive by PCR or RT-PCR/total number of grafted plants							
		GRLaV	GFLV	GRSPaV	HpSVd	GYSVd-1	CEVd	CEYVd	Symptoms
CF - Withsymptoms	CF	30/30	0/30	25/30	30/30	6/30	0/30	0/30	30/30
CF - No symptoms	CF	0/30	0/30	28/30	30/30	5/30	0/30	0/30	0/30
MR - With symptoms	CF	30/30	3/30	22/30	30/30	4/30	0/30	0/30	30/30
MR - No symptoms	CF	0/30	0/30	28/30	30/30	8/30	0/30	0/30	0/30

† CF = Cabernet Franc, MR = Merlot.

* Single-budded, virus-free scion of Cabernet Franc (CF).

GRLaV = Grapevine redleaf-associated virus, GRSPaV = *Grapevine rupestris stem pitting-associated virus*, GFLV = *Grapevine fanleaf virus*, HpSVd = *Hop stunt viroid*, GYSVd-1 = *Grapevine yellow speckle viroid 1*, GYSVd-2 = *Grapevine yellow speckle viroid 2*, CEVd = *Citrus exocortis viroid*, and CEYVd = *Citrus exocortis Yucatan viroid*.

Symptoms and the presence of Grapevine redleaf-associated virus and other viruses and viroids in Cabernet Franc scions grafted onto rootstocks of Merlot and Cabernet Franc with and without GRD symptoms.

### GRLaV is Transmissible by the Virginia Creeper Leafhopper

**Figure 5:**
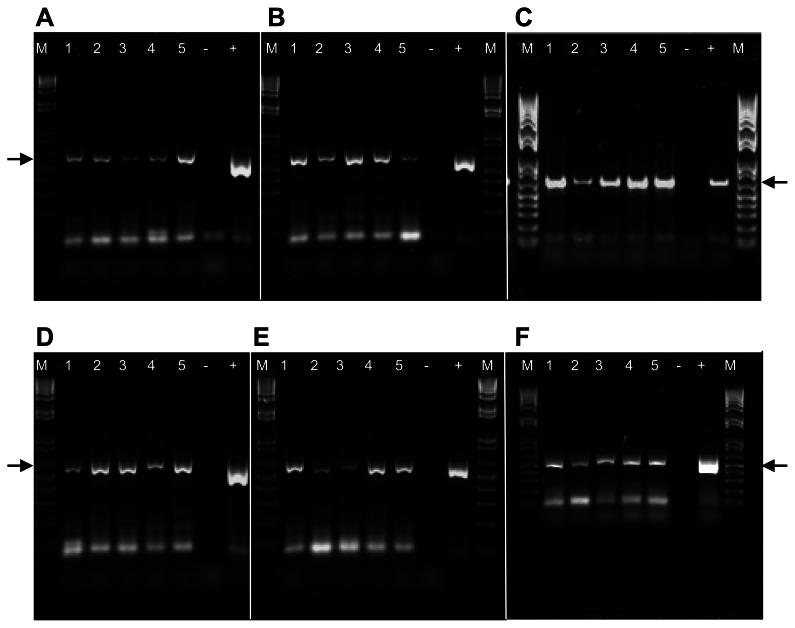
Detection of Grapevine redleaf-associated virus (GRLaV) in grapevines inoculated with viruliferous Virginia creeper leafhoppers. An agarose gel (0.8%) showing amplification of partial IR and CP regions of GRLaV from (A) Cabernet Franc, (B) Chardonnay, (C) Merlot, (D) Pinot Noir, (E) Pixie and (F) Sangiovese. Lanes 1 to 5 represent individual grapevines, ‘+’ represents positive control for GRLaV, ‘−’ represents healthy control negative for GRLaV, and ‘M’ represents 1 kb plus DNA ladder (Invitrogen). Arrows represent ∼550 bp DNA band specific to GRLaV.

**Figure 6:**
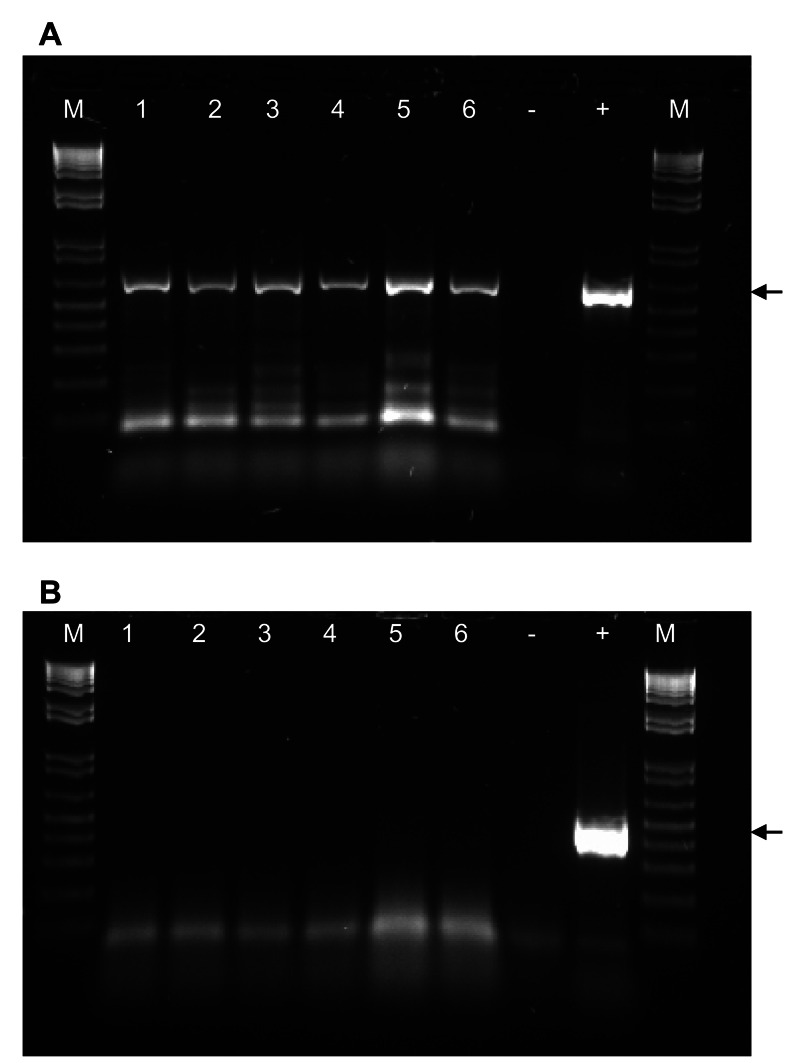
Detection of Grapevine redleaf-associated virus (GRLaV) and ***Grapevine leafroll-associated virus 3*** **(GLRaV-3) in Virginia creeper leafhopper exposed to grapevines mixed infected with GRLaV and GLRaV-3.** An agarose gel (0.8%) showing (A) amplification of partial IR and CP regions of GRLaV and (B) no amplification of GLRaV-3-specific sequence from individual leafhoppers (lanes 1–6) subjected to 72 hr acquisition access on Mourvèdre mixed infected with GRLaV and GLRaV-3. ‘+’ represents positive control for the respective viruses, ‘−’ represents healthy control negative for the respective viruses, and ‘M’ represents 1 kb plus DNA ladder (Invitrogen). Arrow represents ∼550 bp and ∼330 bp DNA band specific to GRLaV (A) and GLRaV-3 (B), respectively.

We investigated the possibility of GRLaV transmission by *Er. ziczac* (Fig. 2), a significant insect pest of wine grapes in Washington vineyards [33]. As shown in Fig. 5, newly emerging leaves of Cabernet Franc, Chardonnay, Merlot, Pinot Noir, Pixie and Sangiovese grapevines inoculated with potentially viruliferous leafhoppers tested positive for GRLaV by PCR using virus-specific primers listed in Table 2. The specificity of amplified DNA fragments was confirmed by cloning and sequencing (data not shown). To ascertain the specificity of GRLaV acquisition by *Er. ziczac*, adult leafhoppers were given a 72 hr AAP on grapevines (cv. Mourvèdre) doubly infected with *Grapevine leafroll-associated virus 3* (GLRaV-3, genus *Ampelovirus*, family *Closteroviridae*) and GRLaV, and transferred onto virus-free Cabernet Franc grapevines for a 72 hr IAP. Newly emerging Cabernet Franc leaves were tested at monthly intervals up to four months post-IAP for GRLaV and GLRaV-3 by PCR and RT-PCR, respectively, using species-specific diagnostic primers (Table 2) [27]. The results showed presence of DNA band specific to GRLaV, but not GLRaV-3, in viruliferous leafhoppers (Fig. 6) and corresponding leafhopper-inoculated grapevines (data not shown), confirming the specificity of *Er. ziczac* in transmitting GRLaV from grapevines mixed-infected with a geminivirus and an ampelovirus. The specificity of GRLaV amplicons was further ascertained by cloning and sequencing (data not shown). These results demonstrated that Virginia creeper leafhopper is a vector of GRLaV. The leafhopper-inoculated grapevines have not shown GRD symptoms under greenhouse conditions even at six months post-inoculation. These grapevines will be planted in the field during the spring of 2013 and monitored for symptom development under field conditions as in the case of grafted grapevines described above.

### GRD Significantly Impacts Grapevine Performance, Fruit Yield and Berry Quality

**Figure 7:**
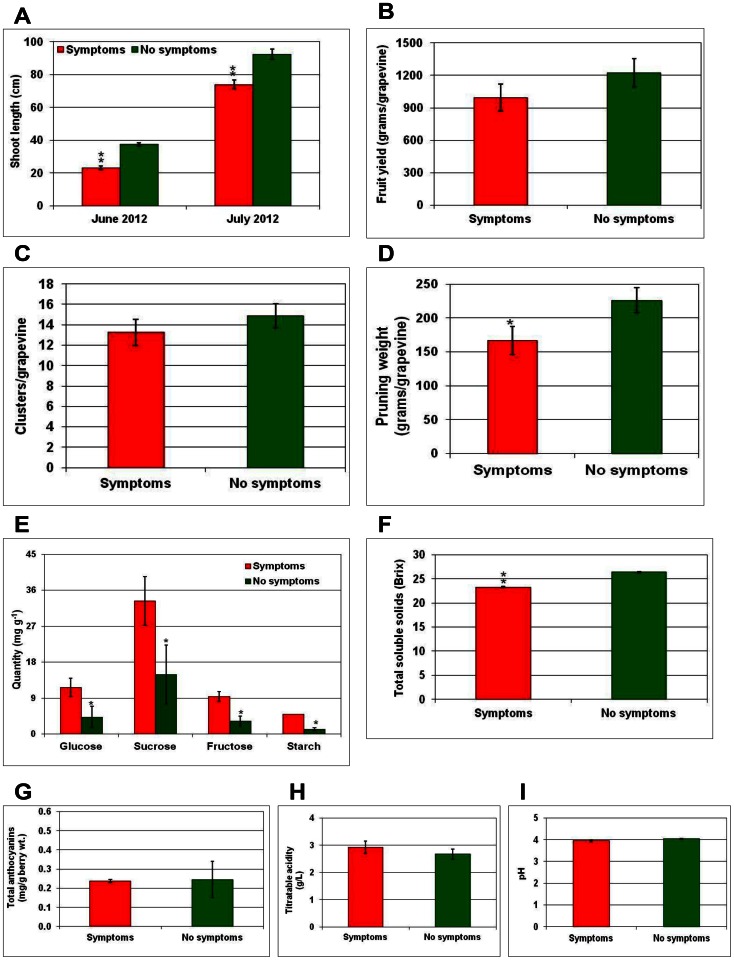
Impacts of Grapevine redleaf disease (GRD) on yield and berry quality parameters for Merlot grapevines. Impacts of the disease on (A) shoot length, (B) fruit yield, (C) number of clusters, (D) pruning weight, (E) leaf carbohydrates, and (F) total soluble solids, (G) anthocyanins, (H) titratable acidity and (I) pH of berries from symptomatic (red color columns) and non-symptomatic (green color columns) grapevines. Columns represent mean value and vertical bars indicate standard errors. Significant differences between symptomatic and non-symptomatic grapevines was determined by one-way ANOVA using the SigmaPlot 11 software and indicated by asterisks (* = p≤0.05 and ** = p≤0.001).

**Figure 8:**
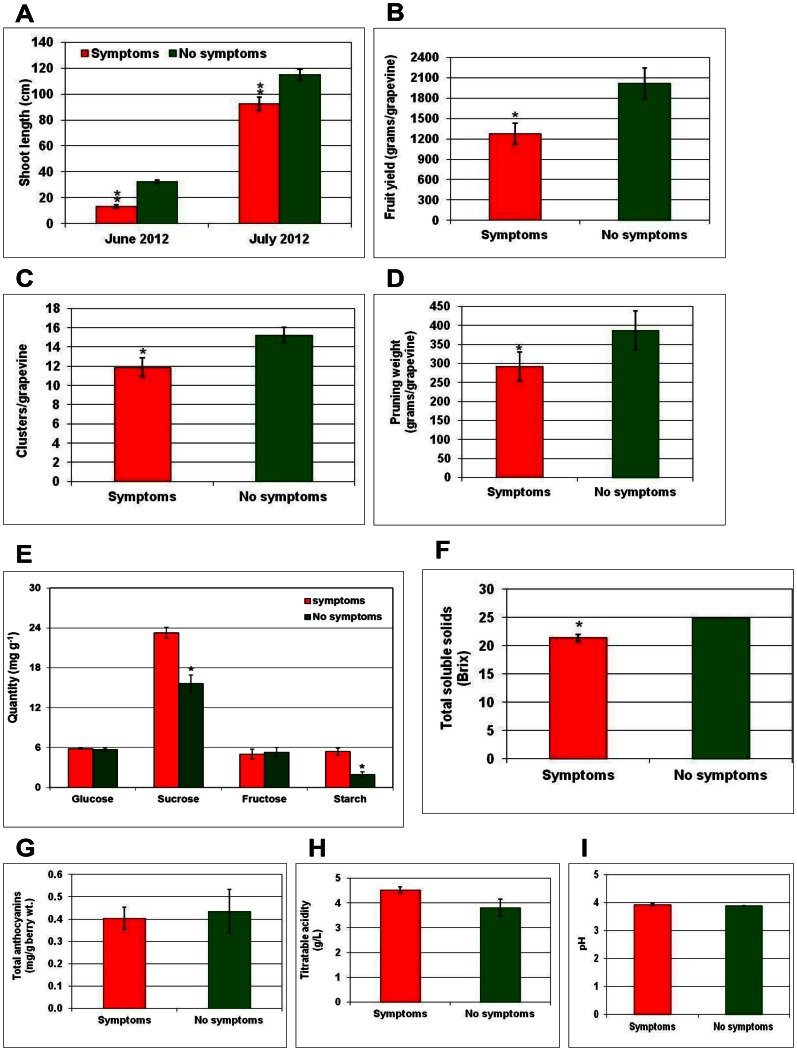
Impacts of Grapevine redleaf disease (GRD) on yield and berry quality parameters for Cabernet Franc grapevines. Impacts of the disease on (A) shoot length, (B) fruit yield, (C) number of clusters, (D) pruning weight, (E) leaf carbohydrates, and (F) total soluble solids, (G) anthocyanins, (H) titratable acidity and (I) pH of berries from symptomatic (red color columns) and non-symptomatic (green color columns) grapevines. Columns represent mean value and vertical bars indicate standard errors. Significant differences between symptomatic and non-symptomatic grapevines was determined by one-way ANOVA using the SigmaPlot 11 software and indicated by asterisks (* = p≤0.05 and ** = p≤0.001).

In order to assess the effects of GRD on vine vigor, the length of growing shoots in own-rooted symptomatic and non-symptomatic Merlot and Cabernet Franc grapevines was measured at two time points (June and July) during early stages of the crop before the grapevines reached 50% bloom during the 2012 season. In the case of Merlot, symptomatic grapevines were 19% and 23% less in average shoot length in June and July, respectively, compared to non-symptomatic grapevines (Fig. 7A). Similarly, symptomatic grapevines of Cabernet Franc were 14% and 18% less in average shoot length compared to non-symptomatic grapevines (Fig. 8A). Yield measurements at the time of commercial harvest indicated that fruit yield was about 22% and 37% less in GRD-affected Merlot and Cabernet Franc grapevines, respectively, compared to non-symptomatic grapevines (Fig. 7B and 8B). This was contributed largely by the lower number of clusters in GRD-affected grapevines than in non-symptomatic grapevines (Fig. 7C and 8C). Total weight of cane prunings per grapevine, a measure of growth during the preceding season, taken in February 2012 was less by 26% in symptomatic grapevines of Merlot (Fig. 7D) and 25% in symptomatic grapevines of Cabernet Franc (Fig. 8D) compared to corresponding non-symptomatic grapevines. The above results indicated negative impacts of GRD on grapevine vigor and fruit yield. Single point estimation of soluble carbohydrates in symptomatic leaves indicated a significantly higher quantity of sucrose and starch, in both cultivars, compared to corresponding non-symptomatic leaves (Fig. 7E and 8E). In the case of Merlot, quantities of sucrose and its hydrolytic products (glucose and fructose) and starch were significantly higher in symptomatic leaves compared to non-symptomatic leaves. In contrast, only sucrose and starch levels were significantly higher in symptomatic than non-symptomatic leaves of Cabernet Franc. These results indicate an accumulation of carbohydrates in leaves showing GRD symptoms with levels of individual carbohydrates showing distinct differences between the two cultivars.

Analysis of fruit quality attributes showed that berries from GRD-affected grapevines of both cultivars were lower in TSS, with an average of 11.7% reduction in Merlot (Fig. 7F) and 13.9% reduction in Cabernet Franc (Fig. 8F), compared to berries from respective non-symptomatic grapevines. In addition, the total amount of berry anthocyanins from GRD-affected grapevines was less by an average of 4% in Merlot (Fig. 7G) and 9% in Cabernet Franc (Fig. 8G), compared to respective non-symptomatic grapevines. In contrast, TA was higher by 9% in GRD-affected Merlot grapevines (Fig. 7H) and 16% higher in Cabernet Franc grapevines (Fig. 7H) compared to respective non-symptomatic grapevines. No difference was observed in pH of juice extracted from berries of symptomatic and non-symptomatic grapevines of either cultivar (Fig. 7I and 8I). Taken together, these results clearly showed a significant negative impact of GRD on grapevine vigor, fruit yield and key attributes of berry quality in own-rooted Merlot and Cabernet Franc cultivars under commercial growing conditions in eastern Washington.

## Discussion

The development of NGS technologies facilitates quick identification of viruses in a variety of samples [34], [35]. As total RNA preparations can contain about 90% of rRNA [36], a single-pass ribo-depletion prior to cDNA library preparation improves the efficiency of high-throughput sequencing for mining known and previously unrecognized pathogens [37]. Hence, ribo-depletion was used to subtract unwanted host cellular RNAs before cDNA library preparation and Illumina sequencing to generate informative sequence reads. This approach has resulted in identification of sequences specific to three distinct viruses (GRLaV, GRSPaV and GFLV) and four viroids (HpSVd, GYSVd-1, CEVd and CEYVd) in grapevines showing GRD symptoms and GRSPaV and all four viroids in non-symptomatic grapevines. Our results (Table 1) also indicated that ribo-depletion coupled with mRNA-depletion of RNA samples resulted in significant loss of informative reads, thus drastically reducing the comprehensiveness of sequence coverage across the genome of a given virus or viroid. Considering that many grapevine viruses and viroids occur in low titer and as mixtures in individual grapevines, the impaired sensitivity of this approach may impede our ability to efficiently capture genome sequences of viruses and viroids. Thus, it can be concluded from our results that a single pass ribo-depletion is sufficient to obtain informative reads and additional mRNA depletion may introduce unnecessary extra handling steps with no real advantage in terms of preserving target sequences for identifying viruses and viroids in an unbiased manner.

In addition to this study, unbiased, culture-independent methods have been successfully used previously in documenting the taxonomic composition of sub-cellular parasites present in grapevines [17], [18], [27], [38]. All these studies highlight the power of NGS strategy in profiling pathogen communities, especially those having low titers in woody perennial crops, for investigating diseases of unknown etiology. Since greater depth and specificity for the detection of viruses and viroids were achieved by these approaches, NGS techniques are also emerging as an efficient alternative to current methods in plant pathogen diagnostics in quarantine and clean plant programs. However, it should be emphasized that the use of molecular and serological diagnostic assays in practical applications cannot be totally replaced with NGS technologies. Rather, the data generated from NGS can be used effectively to improve efficiency and reliability of diagnostic assays in molecular epidemiological investigations and etiological studies for plant health management.

The presence of GRLaV and GFLV in symptomatic grapevines (Table 1) implicates their role in GRD symptoms. Although single infection of GFLV was reported to produce fanleaf disease symptoms in own-rooted wine grape cultivars [26], we have not observed these symptoms in grapevines showing GRD symptoms. A likely explanation could be that GFLV failed to produce symptoms due to antagonistic interactions with other viruses and viroids. In own-rooted cultivars, GRSPaV is known to cause asymptomatic infections when present singly. In general, viroids are ubiquitous in cultivated grapevines causing symptomless infections [8], [9], although it was suggested that GYSVd-1 and GFLV could contribute synergistically in mixed infections to the production of vein-banding and yellow speckle symptoms [11]. Since we did not observe vein-banding and yellow speckle symptoms in either Merlot or Cabernet Franc grapevines, it is likely that these two agents are benign, or GRD symptoms may be masking symptoms specific to synergistic interactions between GFLV and GYSVd-1. Since GRLaV has been consistently found only in grapevines showing GRD symptoms (Fig. 1) and its presence and associated symptoms proved to be graft-transmissible (Table 3), it is reasonable to infer GRLaV as largely responsible for the production of redleaf symptoms in wine grape cultivars Merlot and Cabernet Franc. However, this is circumstantial at present and expression of GRD symptoms may involve multiple interactions between disparate viruses and viroids identified in this study involving antagonistic, synergistic or additive effects [39], [40], [41]. It is likely that intrinsic difference in replication and accumulation of individual viruses and viroids and the efficiency of grapevine RNA silencing machinery in recognizing and targeting their genomes could be contributing to the overall interactions leading to establishment of disease symptoms. Although deciphering individual effects of multiple combinatorial interactions among these disparate agents in grapevine is a challenging task, monitoring grapevines inoculated with GRLaV by Virginia creeper leafhoppers would provide leads in advancing our understanding of the etiology of GRD.

The genome of GRLaV has six predicted ORFs and the nonanucleotide sequence (TAATATT↓AC) at its putative origin of replication that is conserved among most species of the family *Geminiviridae*. However, arrangement of the six ORFs within the genome (Fig. 3) and sequence comparison of the Rep and CP proteins (Fig. S2 and S3) clearly showed distinct differences between GRLaV and other well-characterized geminiviruses. These obvious differences together with a very low degree of genome sequence identity (31% to 44%) between GRLaV and other currently known geminiviruses, including leafhopper-transmitted viruses, would justify GRLaV as a distinct virus species. Based on less than 75% pairwise sequence identity criterion for the demarcation of species in the family *Geminiviridae* [42], [43] and other considerations mentioned above, we propose that GRLaV should be recognized as a new species in this family. Phylogenetic analysis of the complete genome (Fig. 3) and amino acid sequences of the Rep and CP genes (Fig. 4) and the uniqueness of its genome organization further support that GRLaV could represent a new evolutionary lineage within the family *Geminiviridae*. By accommodating these features, we propose to establish a new genus **Graingemvirus**, derived from grapevine-infecting geminivirus, to represent this genus-level geminivirus lineage with GRLaV as the type member. In a recent study, Krenz *et al*. [19] tentatively designated the circular ssDNA virus from grapevines as Grapevine cabernet franc-associated virus (GCFaV), since it was first detected in cv. Cabernet Franc. In California, the same virus was designated as Grapevine red blotch-associated virus, considering the predominant symptom observed in infected red-berried wine grape cultivars [44]. Since grapevine-infecting geminiviruses reported from New York, California, Washington (this study) and British Columbia (Canada) are virtually identical at the genome level, we propose that all of these isolates be renamed as Grapevine redleaf-associated virus. From a practical point of view, the name redleaf disease, instead of red blotch disease, is a broader term to cover the full spectrum of symptoms observed in different red-berried cultivars, so that growers can pay attention to all suspicious red leaves when scouting for this disease in their vineyards. GRD symptoms observed under Washington conditions appear to be similar, though not identical, to grapevine red blotch disease reported from California and New York [45]. It is likely that symptom expression may vary between own-rooted grapevines planted in Washington vineyards and grafted grapevines planted in California and New York. It should be noted, however, that visible symptoms of GRD were observed only in red-berried cultivars, even though the geminivirus can infect both red- and white-berried cultivars [44].

To our knowledge, none of the 615 species of the insect genus *Erythroneura* [46] have so far been reported as vectors of any plant virus. In this context, our finding that *Er. ziczac* can vector GRLaV has important implications in the ecology and epidemiology of the virus. It needs to be determined if other species of leafhoppers, such as the eastern grape leafhopper (*Er. comes* Say), three-banded leafhopper (*Er. tricincta* Fitch), the variegated leafhopper (*Er. variabilis)* and the western grape leafhopper (*Er. elegantula* Osborn), commonly infesting grapevines in different grapevine-growing regions of the USA [47], can transmit GRLaV. Among them, *Er. ziczac* and *Er. elegantula* have been reported as the two primary leafhopper pests in south central Washington vineyards [48]. With piercing and sap sucking mouth parts, nymphal and adult leafhoppers of both species are known to cause feeding damages to grapevines, with *Er. ziczac* being a more efficient and damaging pest than *Er. elegantula* [33], [48]. As the coat protein plays a critical role in vector transmission of geminiviruses and in determining specificity of transmission by individual genera or species of vector [49], studies are being conducted on virus-vector relationships and the ecology of *Er. ziczac* and *Er. elegantula* in relation to the host range of GRLaV for a detailed understanding of the epidemiology of GRD in Washington vineyards.

As a perennial, GRLaV-infected grapevines could serve as a constant source of inoculum for the leafhopper vector to spread the virus to other vineyards in the vicinity. Due to their high mobility and ability to migrate long distances when assisted by wind currents, leafhopper vectors can spread GRLaV rapidly to healthy vineyards located in proximal locations or at a distance to infected blocks leading to virus outbreaks. Our preliminary surveys conducted during 2012 season using diagnostic primers developed in this study (Table 2) indicated that GRLaV is prevalent in several red-berried wine grape cultivars, in addition to Merlot and Cabernet Franc (data not shown). It is likely that these infections were due to virus transmission by viruliferous leafhoppers. In this context, a recent report of an expanded geographic range of *Er. ziczac* to some vineyards of Northern California, from the Oregon border to the Northern Sacramento Valley, Northern Sierra foothills, and Lake and Mendocino Counties (The University of California Cooperative Extension, Napa County; http://cenapa.ucanr.edu/news_970/Vineyard_Views/newsitem=44921) highlight the invasive potential of *Er. ziczac* and its ability to spread GRLaV to other grapevine-growing regions.

Until recently, only RNA viruses have been documented in grapevines, giving the impression that this plant species may be recalcitrant to infection with DNA viruses. However, identification of a badnavirus [50] and a geminivirus [19], [44], [this study], in grapevines indicated that this perennial woody species can also support multiplication of DNA viruses with distinct genomes and replication strategies. Although the origin of GRLaV or its most likely natural host cannot be determined with certainty at this time, it is possible that the virus was originally introduced into *V. vinifera* at the center of its origin in Asia Minor by a competent vector from an indigenous reservoir host plant species or got infected subsequent to domestication and widespread cultivation. It is likely that such a host species ‘jump’ could have contributed to geographic range expansion of the virus to many viticultural areas via dissemination of clonally propagated grapevine cuttings. Recent reports from New York [19], California [44], Washington (this study) and other regions of the USA (our unpublished data) and British Columbia in Canada would support this argument. Consequences of such host species ‘jumps’ and ensuing geographic range expansion of viruses through a variety of avenues have been discussed earlier [14], [51]. Thus, extensive sampling of GRLaV in cultivated and wild species of the genus *Vitis* and possible alternative hosts in different grapevine-growing regions within and outside the USA would provide clues about the evolutionary origin of the virus.

Our results (Fig. 7 and 8) have shown that GRD can cause significant negative impacts on grapevine performance, fruit yield and quality attributes of berries such as sugars and anthocyanins. Significantly higher amounts of sugars in symptomatic leaves (Fig. 7E and 8E) and lower amounts of sugars in berries (Fig. 7F and 8F), compared to respective samples from non-symptomatic grapevines, is most likely a consequence of reduced sugar export from these source leaves to ripening berries. One explanation would be that mature leaves proximal to berry clusters act as a major source of sucrose for ripening berries [52], [53] and disruption of phloem-mediated export of sucrose as the main sugar for translocation of photosynthates destined to these berries could result in retention of sucrose in infected leaves. Such an accumulation of sucrose due to altered source-sink relations is likely to increase expression of cell wall invertase, which hydrolyses sucrose to glucose and fructose that are efficiently retained by the mesophyll cells [54], [55]. Higher sucrose levels due to their retention in mesophyll cells, in turn, could trigger production of anthocyanins leading to redleaf symptoms [56]. In this context, it is worth mentioning that symptoms of GRD and grapevine leafroll disease [3] appeared only after *véraison* and symptoms of both diseases overlapped to a greater extent in several red-berried cultivars. Thus, further comparative studies are warranted to better understand the underlying molecular and physiological events contributing to similar, though not identical, symptoms in two disparate pathosystems. In addition, using diagnostic primers specific to GRLaV and grapevine leafroll-associated viruses, it is now possible to elucidate epidemiological differences between GRD and grapevine leafroll disease for practical applications to manage these two distinct diseases in vineyards.

## Supporting Information

Figure S1 **Alignment of mapped contigs to respective genomes of Grapevine redleaf-associated virus, Grapevine fanleaf virus, Grapevine rupestris stem pitting-associated virus, Hop stunt viroid, Grapevine yellow speckle viroid 1, Citrus exocortis viroid and Citrus exocortis Yucatan viroid.** Nucleotide numbers of each virus and viroid genome is indicated at the top and bottom. Each bar represents the location of individual contigs aligning with the genome.
(PDF)

Figure S2 **Multiple alignment of predicted amino acid sequences of partial replication-associated proteins (Rep) of Grapevine redleaf-associated virus (GRLaV) and representatives of approved genera in the family** ***Geminiviridae*** **.** Name of each genus is listed on the right and corresponding amino acid sequence of individual viruses within each genus is listed on the left in the same color. Note that only the abbreviation for each virus with corresponding accession number in the parenthesis is listed. Conserved or unique amino acid motifs are highlighted in different colors. Rolling circle replication motifs I–III and the GRS motif [31] are highlighted in yellow. Percentage (%) identities shared by all viruses in each of these motifs are shown in parenthesis. The retinoblastoma-like protein binding sequence (RBR) is highlighted in dark green. Additional motifs of unknown functions are highlighted in light blue color.
(PDF)

Figure S3 **Multiple alignment of predicted amino acid sequence of the coat protein of Grapevine redleaf-associated virus (GRLaV) and representatives of approved genera in the family** ***Geminiviridae*** **.** Name of each genus in the family is listed on the right and corresponding amino acid sequence of individual viruses within each genus is listed on the left in the same color. Note that only the abbreviation for each virus with corresponding accession number in the parenthesis is listed. Amino acid motifs conserved (light green) and unique (yellow, orange and grey) to specific genera are highlighted. Motif in yellow was determined to be critical for leafhopper transmission of *Beet mild curly top virus* [57] and the motif in orange is a conserved zinc finger motif in begomoviruses [58]. The motif of unknown function is highlighted in grey color.
(PDF)

Table S1 **Classification of sequence reads.** Classification and abundance of high-throughput sequence reads obtained from ribo-depleted and double depleted cDNA libraries derived from symptomatic and non-symptomatic grapevine leaves, when mapped to virus/viroid database.
(PDF)
