# Correction: A Leafhopper-Transmissible DNA Virus with Novel Evolutionary Lineage in the Family *Geminiviridae* Implicated in Grapevine Redleaf Disease by Next-Generation Sequencing

**DOI:** 10.1371/journal.pone.0147510

**Published:** 2016-01-15

**Authors:** Sudarsana Poojari, Olufemi J. Alabi, Viacheslav Y. Fofanov, Rayapati A. Naidu

Table 2 is missing information regarding GVGF and GVGR. Please see the corrected Table 2 here.

**Table 2:** Oligonucleotide primers used for amplification of virus- and viroid-specific sequences by RT-PCR and PCR.

Name	Sequence	Genome Position (from 5’end)
**For genome characterization of GRLaV:**		
GRLaV-1-For	CCCATGGTACGTGGTATTCTTGCG	11–34
GRLaV-1-Rev	CAGTTCCAGTAGGAAACCGATC	1033–1054
GRLaV-2-For	GGCCTCGTAGTAGGCCTTGTC	811–831
GRLaV-2-Rev	GCAACATTCAAGCCGTGGGCTG	1967–1988
GRLaV-3-For	GCATAGTCCAGACAGTCGTTGTAC	1728–1751
GRLaV-3-Rev	GCCCAGAGATGTCGCCGACGTGC	2778–2800
GRLaV-4-For	GTAGATTGAGGACGTATTGG	2601–2620
GRLaV-4-Rev	CGCAAGAATACCACGTACCATGGG	34–11
**For detection of viruses and viroids by PCR/RT-PCR:**		
GVGF^1^	CTCGTCGCATTTGTAAGA	255–272
GVGR^1^	ACTGACAAGGCCTACTACG	793–811
GFLV-For	ACTGGTTTGACGTGGGTGAT	2224–2243 (RNA-2)
GFLV-Rev	CCAAAGTTGGTTTCCCAAGA	2526–2545 (RNA-2)
GRSPaV-For	GATGAGGTTCAGTTGTTTC	4372–4390
GRSPaV-Rev	TCACCAAATGTGAGAGTGAGCTG	4771–4793
HpSVd-For	GAGCCCCGGGGCAACTCTTCTC	74–95
HpSVd-Rev	TTTCTCAGGTAAGTACCTCCCTG	50–72
GYSVd-1-For	TGCCTCCGCTAGTCGAGCGG	254–273
GYSVd-1-Rev	CGACGACGAGGCTCACT	88–104
CEVd/CEYVd-For	GGAAACCTGGAGGAAGGTG	9–27
CEVd/CEYVd Rev	CCGGGTACATATTCACCGCGGCA	206–228

GRLaV = Grapevine redleaf-associated virus, GRSPaV = *Grapevine rupestris stem pitting-associated virus*, GFLV = *Grapevine fanleaf virus*, HpSVd = *Hop stunt viroid*, GYSVd-1 = *Grapevine yellow speckle viroid 1*, GYSVd-2 = *Grapevine yellow speckle viroid 2*, CEVd = *Citrus exocortis viroid*, CEYVd = *Citrus exocortis Yucatan viroid*, For = forward (sense) primer, Rev = reverse (antisense) primer.

^1^ Primers provided by Dr. M. R. Sudarshana, Research Biologist, USDA-ARS, Davis, CA 95616.

There is an error regarding the presence of Citrus exocortis yucatan viroid (CEYVd) and Citrus exocortis viroid (CEVd) sequences. The sequences of Citrus exocortis viroid (CEVd) and Citrus exocortis yucatan viroid (CEYVd) originally listed in the NCBI database are wrongly annotated. Citrus exocortis viroid and Citrus exocortis Yucatan viroid are mitochondrial rRNA sequences. NCBI has removed CEVd (accession no. KC427103, KC427104) and CEYVd (accession no. KC427105, KC427106) sequences from GenBank.

Table 1 and S1 Fig reference the incorrect CEVd and CEYVd sequences. Please see the corrected Table 1 and S1 Fig here.

**Table 1:** Classification of pathogen-specific sequence reads from symptomatic and non-symptomatic samples.

Category of sequence reads	Reads from ribo-depleted cDNA library		Reads from dual-depleted cDNA library	
	Symptoms	No Symptoms	Symptoms	No Symptoms
**Reads mapped to specific virus**				
GRLaV	15,036	0	406	0
GFLV	14,383	0	978	0
GRSPaV	662	1,353	42	68
**Reads mapped to specific viroid**				
GYSVd-1	13,914	9,004	987	490
HpSVd	1964	2980	164	185
Others*	8,029	2,931	655	1,616
**Total**	53,988	16,268	3,232	2,359

GRLaV = Grapevine redleaf-associated virus, GRSPaV = Grapevine rupestris stem pitting-associated virus, GFLV = Grapevine fanleaf virus, HpSVd = Hop stunt viroid, GYSVd-1 = Grapevine yellow speckle viroid 1.

*Reads mismapped to virus- or viroid-like sequences.

Classification and abundance of high-throughput sequence reads obtained from ribo-depleted and dual-depleted cDNA libraries were determined by mapping the reads onto the virus/viroid database using mapping software BWA 0.6.

## Supporting Information

S1 Fig Alignment of mapped contigs to respective genomes of Grapevine redleaf-associated virus, Grapevine fanleaf virus, Grapevine rupestris stem pitting-associated virus, Hop stunt viroid, Grapevine yellow speckle viroid 1, Citrus exocortis viroid and Citrus exocortis Yucatan viroid. Nucleotide numbers of each virus and viroid genome is indicated at the top and bottom. Each bar represents the location of individual contigs aligning with the genome.
(TIF)
