# Supplementary figures and images for: A Leafhopper-Transmissible DNA Virus with Novel Evolutionary Lineage in the Family Geminiviridae Implicated in Grapevine Redleaf Disease by Next-Generation Sequencing
Source: PLoS One. 2013 Jun 5;8(6):e64194. doi: 10.1371/journal.pone.0064194 (PMC3673993; doi:10.1371/journal.pone.0064194)

Figure S1

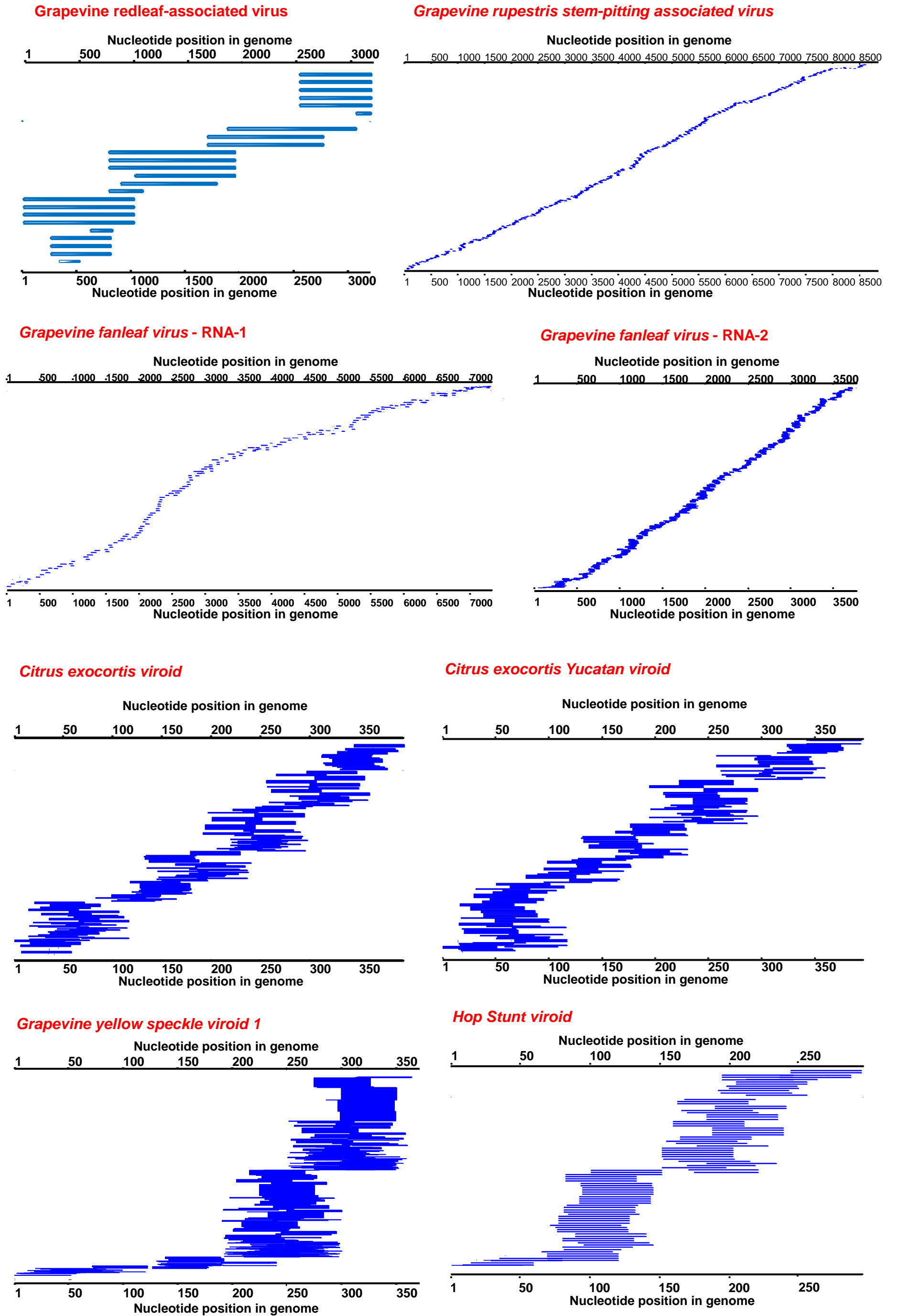

Supplement: Figure S1 — Alignment of mapped contigs to respective genomes of Grapevine redleaf-associated virus, Grapevine fanleaf virus, Grapevine rupestris stem pitting-associated virus, Hop stunt viroid, Grapevine yellow speckle viroid 1, Citrus exocortis viroid and Citrus exocortis Yucatan viroid. Nucleotide numbers of each virus and viroid genome is indicated at the top and bottom. Each bar represents the location of individual contigs aligning with the genome. (PDF) [file pone.0064194.s001.pdf]
