# Supplementary material for: A Leafhopper-Transmissible DNA Virus with Novel Evolutionary Lineage in the Family Geminiviridae Implicated in Grapevine Redleaf Disease by Next-Generation Sequencing
Source: PLoS One. 2013 Jun 5;8(6):e64194. doi: 10.1371/journal.pone.0064194 (PMC3673993; doi:10.1371/journal.pone.0064194)
Supplement: Figure S2 — Multiple alignment of predicted amino acid sequences of partial replication-associated proteins (Rep) of Grapevine redleaf-associated virus (GRLaV) and representatives of approved genera in the family Geminiviridae . Name of each genus is listed on the right and corresponding amino acid sequence of individual viruses within each genus is listed on the left in the same color. Note that only the abbreviation for each virus with corresponding accession number in the parenthesis is listed. Conserved or unique amino acid motifs are highlighted in different colors. Rolling circle replication motifs I–III and the GRS motif [31] are highlighted in yellow. Percentage (%) identities shared by all viruses in each of these motifs are shown in parenthesis. The retinoblastoma-like protein binding sequence (RBR) is highlighted in dark green. Additional motifs of unknown functions are highlighted in light blue color. (PDF) [file pone.0064194.s002.pdf]

Figure S2

```
                                                               Motif I (60%)
                      ....|....|....|....|....|....|....|....|....|....|....|....|....|....|....|....|....|....|....|....|
                          5        15        25        35        45        55        65        75        85        95       105       115
BCTV-G [EU586260]     -------------------------------------------MPRRPT------------FQIRAKNIFLTYPRCSVT-KDDALEILKNIP-CPSDKLFIRVSQEKHQ------DGSLH
BCTV-G [EU193175]     -------------------------------------------MPRRPT------------FQIRAKNIFLTYPRCSVI-KDDALEILKNIP-CPSDKLFIRVSQEKHQ------DGSLH
BCTV-G [HQ634913]     -------------------------------------------MPRRPT------------FQIRAKNIFLTYPRCSVT-KDDALEILKNIP-CPSDKLFIRVSQEKHQ------DGSLH
BCTV-G [HQ214016]     -------------------------------------------MPRRPT------------FQIRAKNVFLTYPRCSII-KEDALEILKNIP-CPSDKLFIRVSQEKHQ------DGSLH
BCTV-G [EU586261]     -------------------------------------------MPRRPT------------FQIRVKNVFLTYPRCSII-KEDALEILKNIP-CPSDKLFIRVSQEKHQ------DGSLH
BCTV-F [AY134867]     -------------------------------------------MPRSPS------------FQIKAKNIFLTYPRCSII-KEDALEILKNIP-CPSDKLFIRVSQEKHQ------DGSLH
BCTV-F [U56975]       -------------------------------------------MPRSPS------------FQIKAKNIFLTYPRCSVI-KEDALEILKNIP-CPSDKLFIRVSQEKHQ------DGSLH
BCTV-E [EU921828]     -------------------------------------------MPRSPS------------FQIRAKNVFLTYPRCSII-KEDALEILKNIP-CPSDKLFIRSFQEKHK------DGTLH
BCTV-D [FJ545686]     -------------------------------------------MS----------------FYKKAKNFFLTYPRCSVT-KEDALEHLLAIP-RPSNKKYIRVSRELHE------DGALH
BCTV-A [AY548948]     -------------------------------------------MP----------------FYRKAKNYFLTYPQCSIS-KEEALEQLLAIS-TPSNKKYIRISRELHD------NGEPH
BCTV-P [EF501977]     -------------------------------------------MP----------------FYKKAKNFFLTYPQCSVT-KEDALEQLLAIN-TPSNKKYIRICRELHE------NGEPH
BCTV-C [X97203]       -------------------------------------------MP----------------FYKKAKNFFLTYPQCSVT-KEDALEQLLAIN-TPSNKKYIRICRELHD------NGEPH
BCTV-C [U02311]       -------------------------------------------MP----------------FYKKAKNFFLTYPQCSVT-KEDALEQLLAIN-TPSNKKYIRICRELHE------NGEPH
TCTV [GU456685]       -------------------------------------------MPRTPRQ-----------YRIHAKNIFLTYPHCHLT-KEDALLQLQTIQ-CPSTKKFIRICRERHD------NGEPH
DoYMV [AM157413]      --------------------------------------------MRTPG------------FRISARNIFLTYPKCSLS-KEQALEQLCRIE-CPSDKLFIRVAQEAHQ------DGTMH
BGYMV [D00201]        -------------------------------------------MPPPQR------------FRVQSKNYFLTYPRCTIP-KEEALSQLQKIH-TTTNKKFIKVCEERHD------NGEPH
MaMPRV [AY044133]     -------------------------------------------MPPPKR------------FRVSSKNYFLTYPRCSIS-KEETLSQLLNIQ-TPTDKKYIKICRELHE------DGQPH
BCTV-H [AF379637]     -------------------------------------------MPPTKR------------FRIQAKNIFLTYPQCSLS-KEEALEQIQGIQ-LSSNKKYIKIARELHE------DGQPH
BCTV-H [M24597]       -------------------------------------------MPPTKR------------FRIQAKNIFLTYPQCSLS-KEEALEQIQRIQ-LSSNKKYIKIARELHE------DGQPH
TGMV [K02029]         ---------------------------------------------------------------MNAKNYFLTYPQCSLS-KEESLSQLQALN-TPINKKFIKICRELHE------DGQPH
TPCTV [X84735]        -------------------------------------------MPSNPKR-----------FQIAAKNYFLTYPNCSLS-KEEALDQLQRLQ-TPTNKKYIKVARELHE------NGEPH
SPLCV [AF104036]      -------------------------------------------MAPPKR------------FKIQAKNYFITYPRCSLS-KEDCLAQLLNIQ-TPSNKKYIHVARELHE------DGEPH
CGMV [AF029217]       -------------------------------------------MPPRSGA-----------FRVNAKNIFATYPRCSLP-KEEALELLRQIP-TAVNKKFIKVARELHE------DGEPH
TLCJV [AB100304]      -------------------------------------------MAPPKR------------FLINAKNYFLTYPQCSLT-KEEALSQLQNLN-TPTNKKFIKICRELHE------DGSPH
CIGMV [DQ641692]      -------------------------------------------MPPPTK------------FRINAKNYFLTYPQCSLT-KEETLARLLAIV-TPTNKKFIKICRELHE------DGSPH
TLCYV [AJ512761]      -------------------------------------------MAPPKR------------FKIQAKNYFLTYPQCSLN-KEEALSQLQALE-TPTNKKYIKICRELHE------DGSPH
HYVV [AB236325]       -------------------------------------------MAPPKR------------FKINSKNYFLTYPHCSLT-KEEALSQLQALE-TPTNKLFIKICRELHE------DGSPH
TYLCCV [AF311734]     -------------------------------------------MAPPNK------------FRINAKNYFLTYPHCSLT-KEEALSQIKTLE-TPVNKLFIRICRELHE------DGSPH
TLCNDV [U15015]       -------------------------------------------MASPRR------------FRVNAKNYFLTYPKCSLT-KEEALSQLQTLE-TPTKKKFIKICRELHE------DGSPH
CLCRV [AM501481]      -------------------------------------------MPPKRN-------------GIYSKNYFITYPKCSLT-KEEALSQLLNIQ-TPTPKKYIRICRELHE------DGTPH
TLCSV [AY044137]      -------------------------------------------MAPPKR------------FKINAKNYFLTYPQCSLS-LSEALSQLQTLE-TPVNKLYIKICRELHE------NGEPH
TYLCSV [X61153]       -------------------------------------------MPRSGR------------FSIKAKNYFLTYPKCDLT-KENALSQITNLQ-TPTNKLFIKICRELHE------NGEPH
ACMV [J02057]         --------------------------------------------MRTPR------------FRIQAKNVFLTYPKCSIP-KEHLLSFIQTLS-LQSNPKFIKICRELHQ------NGEPH
CoGMV [EU636712]      ---------------------------------------------MSGR------------FRKQGVSFFLTWPKCPVT-KESAIHQILALA-LPTNIIYIRVCEEKHQ------DGSPH
TSLCV [AF130415]      -------------------------------------------MPRNPNL-----------FRLAAKNIFLTYPQCDIP-KDEAIEMLQNLPWSVVKPTYIRVAREEHA------DGFPH
SpSCTAV [GU734126]    -------------------------------------------MPRQNKS-----------FRLSSKNIFLTYPQCDIP-KDEVLQLLQNLPWSVVKPTYIRVASERHA------DGTPH
HrCTV [U49907]        -------------------------------------------MPRQPNS-----------FRIQARNIFLTYPQCTIP-KDEMLQLLQNLNWTVVKPTYIRVAAERHA------DGTPH
ECSV [FJ665632]       ------------------------------------------MASSSHR------------FRIQGRAFFLTYSQCPRE-PKDVGEFLTSHSTLASHVVYVRVQQEKHQ------DGNNH
GRLaV-WA [JQ000000]   -------------------------------------------MASSSS------------FNLRSKNFFLTYPQCPVH-KQFALDFFKSKF--QKTLDYALIGEEKHQ------NGEPH
GCFaV-NY [JQ901105]   -------------------------------------------MASSSS------------FNLRSKNFFLTYPQCPVH-KQFALDFFKSKF--HKTLDYALIGEEKHQ------NGEPH
BCTIV [JQ707938]      -------------------------------------------MPRAPNRNRPTPNQPGY-LRFQKKNAFLTYSQIGGDFKDYIFEKLKTLL-ESYVILFLAVSLEHHQPTEEQPEGGFH
BCTIV [JQ707939]      -------------------------------------------MPRAPNRNRPTPNQPGY-LRFQKKNAFLTYSQIGGDFKDYIFEKLKTLL-ESYVILFLAVSLEHHQPTEEQPEGGFH
BCTIV [EU273816]      -------------------------------------------MPRAPNRNRPTPNQPGY-LRFQKKNAFLTYSQIGGDFKDYIFEKLKTLL-ESYVILFLAVSLEHHQPTEEQPEGGFH
BCTIV [JQ707945]      -------------------------------------------MPRAPNRNRPTPNQPGY-LRFQKKNAFLTYSQIGGDFKDYIFEKLKTLL-ESYVILFLAVSLEHHQPTEEQPQGGFH
SCTAV [HQ443515]      -------------------------------------------MARTPSRNRPNQNGPGY-FRFQKKNAFLTYSQIGGDFKDYIFEKLLALL-QQFEVLFLSVALEHHQPTESNPEGGFH
TbYDV [M81103]        -------------------------------------------MPSAPQKTKS--------FRLQTKYVFLTYPRCSSS-AENLRDFLWDKL-SRFAIFFIAIATELHQ------DGTPH
CpYV [JN989439]       -------------------------------------------MPSPSKKSPS--------FRLQTKYVFLTYPHCSSS-AEGLRDFLWDKL-SRFAIFFIAIATELLP---------AH
CpRV [GU256532]       -------------------------------------------MPRLNKKTSN--------FRFQSKYVFLTYPHCNSN-PEALRDYLWEKL-TRFIIFFIAVASEVHQ------DGSPH
CpCDV [AM850136]      -------------------------------------------MPSANKN-----------FRFQSKYVFLTYPKCSSP-RDDLFEFLWEKL-TPFLIFFLGVATELHQ------DGTTH
ODV [AM296025]        ---------------------------------------MATVASSSTR------------FRVYSKYLFLTYPQCILE-PQYALDSLRSLL-QKYKPLYICSVRERHE------DNSPH
WDV [X02869]          -----------------------------------------MASSSAPR------------FRVYSKYLFLTYPECTLE-PQYALDSLRTLL-NKYEPLYIAAVRELHE------DGSPH
BCSMV [HQ113104]      ---------------------------MASFVSETSDARGQTGAPRSPSGEVGAPGAVAACFEVRSRNIFLTYSKCHLD-PVFMQEHLSSLL-RRFEPTYVYVAREEHQ------DGSYH
PSMV [JF905486]       ---------------MSSLVSETSNSEVGSQMESPGRGGQSIDAPSSSC------------FKVRARNLFLTYSKCNLT-AVFLLEYISSLL-KKYCPTYIYVAQEAHK------DGSHH
DDSMV [HM122238]      ------------------------MSSQLVSDSVMFDPRSYGEYPSSESAASLPLS-----FNVRSQHVFLTYPRCPIP-PKDAGSFLKKLC-KRYNIQYMYIAQELHQ------DGEPH
CSMV [M20021]         -------------------MPVSESEGEGSGTSVQVPSRGGQVTPGEKA------------FSLRTKHVFLTYPRCPIS-PEEAGQKIADRL-KNKKCNYIYISREFHA------DGEPH
MiSV [D01030]         -----------------------------------------MRAPASSAASNRPGPSNHPTPRWNSKQFFLTYPHCNLT-PSELMKELFSRL-TEKIPGYIKVSQEFHK------DGDPH
EMSV [JF508490]       ---------------------------------MSQDQDTLGSSSDGSR------------FRISSKQLFLTYPRCDLS-PKDLGLELLQLL-IQNKPKYIHVTQELHK------DGFPH
MRSV [JQ624880]       MSAFGNHFVHMPSVQAGVFNPMPGSDYPSEEEDLHQQNTPVGPDPPRRN------------FQFKSANAFLTYPRCLLT-PFEAGQHLWEVA-RHWTPSYVLASSESHQ------DGTPH
DSV [M23022]          -------------------------------------------MAANRS------------FRHRNANTFLTYSKCDHS-PQLIADHLWDLL-KSWNPIYILVASEHHA------DGSLH
MSV [AF329881]        ----------------------------------------MASSSNRQ------------FSHRNANTFLTYPKCPEN-PEIACQMIWELV-VRWIPKYILCAREAHQ------DGSLH
PanSV [L39638]        --------------------------------MSTSLSITSDGRHSVRS------------FRHRNANTFLTYSKCPLE-PEFIGEHLFRLT-KDFEPAYILVVRETHQ------DGTWH
SacSV [GQ273988]      ---------------------------------MAYANSTSTESNSSRS------------FRHRNANTFLTYSKCSLD-PEILGLSLWSKL-APWTPAYILVAREAHQ------DGTWH
USV [EU445697]        --------MGWVGFRRELSRHRVWGEQIAPRLELMATVGSSSNSVASRS------------FKHRNANTYLTYPKCPLE-PEAIGLTLWSLI-APWEPAYIIVCREAHQ------DGTWH
SSV [M82918]          ----------------------------------MSTVGSTVSSTPSRR------------FKHRNVNTFLTYSRCPLE-PEAVGLHIWSLI-AHWTPVYVLSVRETHE------DGGYH
SSRV [AF072672]       -------------------------------------------MPSQEDSTVASRP-----FKHRNANTFLTYSRCRLD-PEAVGLILWQLI-SHWSPAYILVSREAHA------DGEWH
ESV [EU244915]        ------------------------------------MSSIASTVPSAPTRR----------FKHRNVNTFLTYPHCTLE-PEVVGLVLWSLL-ESWTPAYIVVSREAHQ------DGSWH
SSEV [AF239159]       ----------------------------------MTTVGSAESGSAIRS------------FKHRNVNTFLTYPKCHLE-PEAVGLHLWSLI-GHWNPAYIVVSREAHA------DGSWH
```

Curtovirus

Turncurtovirus

Topocuvirus

Begomovirus

Eragrosvirus

Graingemvirus

Becurtovirus

Mastrevirus

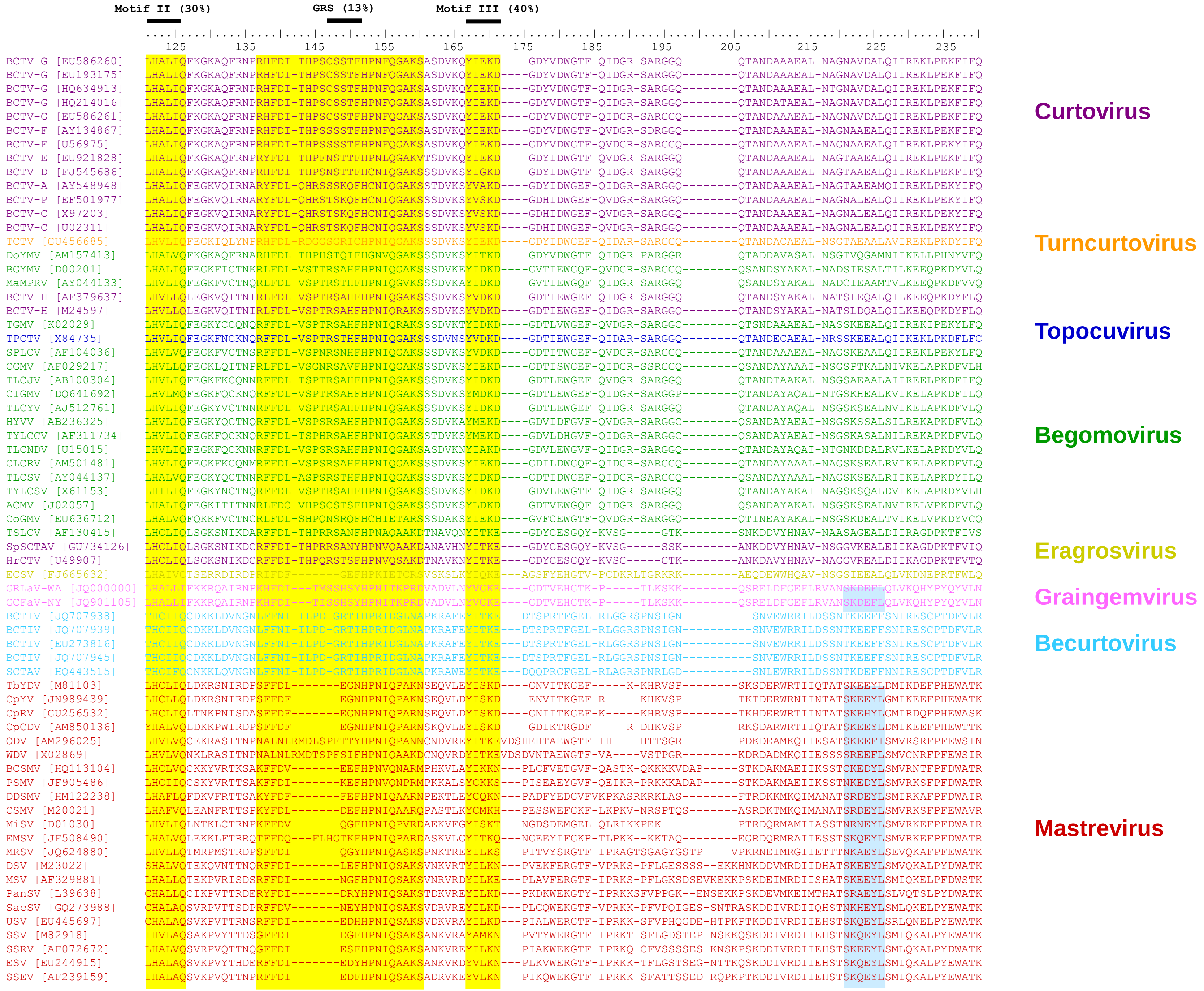

Motif II (30%)
GRS (13%)
Motif III (40%)
125 135 145 155 165 175 185 195 205 215 225 235
BCTV-G [EU586260] LHALIQFKGKAQFRNPRHFDI-THPSCSSTFHPNFQGAKSASDVKQYIEKD----GDYVDWGTF-QIDGR-SARGGQ--------QTANDAAAEAL-NAGNAVDALQIIREKLPEKFIFQ
BCTV-G [EU193175] LHALIQFKGKAQFRNPRHFDI-THPSCSSTFHPNFQGAKSASDVKQYIEKD----GDYVDWGTF-QIDGR-SARGGQ--------QTANDAAAEAL-NAGNAVDALQIIREKLPEKFIFQ
BCTV-G [HQ634913] LHALIQFKGKAQFRNPRHFDI-THPSCSSTFHPNFQGAKSASDVKQYIEKD----GDYVDWGTF-QIDGR-SARGGQ--------QTANDAAAEAL-NTGNAVDALQIIREKLPEKFIFQ
BCTV-G [HQ214016] LHALIQFKGKAQFRNPRHFDI-THPSCSSTFHPNFQGAKSASDVKQYIEKD----GDYVDWGTF-QIDGR-SARGGQ--------QTANDATAEAL-NAGNAVDALQIIREKLPEKFIFQ
BCTV-G [EU586261] LHALIQFKGKAQFRNPRHFDI-THPSCSSTFHPNFQGAKSASDVKQYIEKD----GDYVDWGTF-QIDGR-SARGGQ--------QTANDAAAEAL-NAGNAVDALQIIREKLPEKFIFQ
BCTV-F [AY134867] LHALIQFKGKAQFRNPRHFDI-THPSSSSTFHPNFQGAKSASDVKQYIEKD----GDYVDWGTF-QVDGR-SDRGGQ--------QTANDAAAEAL-NAGNAAEALQIIREKLPEKFIFQ
BCTV-F [U56975] LHALIQFKGKAQFRNPRHFDI-THPSSSSTFHPNFQGAKSASDVKQYIEKD----GDYVDWGTF-QVDGR-SARGGQ--------QTANDAAAEAL-NAGNAAEALQIIREKLPEKYIFQ
BCTV-E [EU921828] LHALIQFKGKAQFRNPRYFDI-THPFNSTTFHPNLQGAKVTSDVKQYIEKD----GDYIDWGTF-QVDGR-SARGGQ--------QTANDAAAEAL-NAGTAAEALQIIREKLPEKFIFQ
BCTV-D [FJ545686] LHALIQFKGKAQFRNPRHFDI-THPSNSTTFHCNIQGAKSSSDVKSYIGKD----GDYIDWGTF-QIDGR-SARGGQ--------QTANDAAAEAL-NAGTAAEALQIIREKLPEKFIFQ
BCTV-A [AY548948] LHALIQFEGKVQIRNARYFDL-QHRSSSKQFHCNIQGAKSSTDVKSYVAKD----GDYIDWGEF-QIDGR-SARGGQ--------QTANDAAAEAL-NAGTAAEAMQIIREKLPEKYIFQ
BCTV-P [EF501977] LHALIQFEGKVQIRNARYFDL-QHRSTSKQFHCNIQGAKSSSDVKSYVSKD----GDHIDWGEF-QVDGR-SARGGQ--------QTANDAAAEAL-NAGNALEALQIIREKLPEKYIFQ
BCTV-C [X97203] LHALIQFEGKVQIRNARYFDL-QHRSTSKQFHCNIQGAKSSSDVKSYVSKD----GDHIDWGEF-QVDGR-SARGGQ--------QTANDAAAEAL-NAGNALEALQIIREKLPEKYIFQ
BCTV-C [U02311] LHALIQFEGKVQIRNARYFDL-QHRSTSKQFHCNIQGAKSSSDVKSYVSKD----GDHIDWGEF-QVDGR-SARGGQ--------QTANDAAAEAL-NAGNALEALQIIREKLPEKYIFQ
TCTV [GU456685] LHVLIQFEGKIQLYNPRHFDL-RDGGSGRICHPNIQGAKSSSDVKSYIEKD----GDYIDWGEF-QIDAR-SARGGQ--------QTANDACAEAL-NSGTAEAALAVIREKLPKDYIFQ
DoYMV [AM157413] LHALVQFKGKAQFRNARHFDL-THPHSTQIFHGNVQGAKSSSDVKSYITKD----GDYVDWGTF-QIDGR-PARGGR--------QTADDAVASAL-NSGTVQGAMNIIKELLPHNYVFQ
BGYMV [D00201] LHALIQFEGKFICTNKRLFDL-VSTTRSAHFHPNIQGAKSSSDVKEYIDKD----GVTIEWGQF-QVDGR-SARGGQ--------QSANDSYAKAL-NADSIESALTILKEEQPKDYVLQ
MaMPRV [AY044133] LHVLIQFEGKFVCTNQRLFDL-VSPTRSTHFHPNIQGVKSSSDVKAYIDKD----GVTIEWGQF-QIDGR-SARGGQ--------QSANDSYAKAL-NADCIEAAMTVLKEEQPKDFVVQ
BCTV-H [AF379637] LHVLLQLEGKVQITNIRLFDL-VSPTRSAHFHPNIQGAKSSSDVKSYVDKD----GDTIEWGEF-QIDGR-SARGGQ--------QTANDSYAKAL-NATSLEQALQILKEEQPKDYFLQ
BCTV-H [M24597] LHVLLQLEGKVQITNIRLFDL-VSPTRSAHFHPNIQRAKSSSDVKSYVDKD----GDTIEWGEF-QIDGR-SARGGQ--------QTANDSYAKAL-NATSLDQALQILKEEQPKDYFLQ
TGMV [K02029] LHVLIQFEGKYCCQNQRFFDL-VSPTRSAHFHPNIQRAKSSSDVKTYIDKD----GDTLVWGEF-QVDGR-SARGGC--------QTSNDAAAEAL-NASSKEEALQIIREKIPEKYLFQ
TPCTV [X84735] LHVLIQFEGKFNCKNQRFFDL-VSPTRSTHFHPNIQGAKSSSDVNSYVDKD----GDTIEWGEF-QIDAR-SARGGQ--------QTANDECAEAL-NRSSKEEALQIIKEKLPKDFLFC
SPLCV [AF104036] LHVLVQFEGKFVCTNSRFFDL-VSPNRSNHFHPNIQGAKSSSDVKSYVDKD----GDTITWGEF-QVDGR-SARGGQ--------QTANDAAAEAL-NAGSKEAALQIIREKLPEKYLFQ
CGMV [AF029217] LHVLLQFEGKLQITNPRLFDL-VSGNRSAVFHPNIQGAKSSSDVKSYIEKD----GDTISWGEF-QIDGR-SSRGGQ--------QSANDAYAAAI-NSGSPTKALNIVKELAPKDFVLH
TLCJV [AB100304] LHVLIQFEGKFKCQNNRFFDL-TSPTRSAHFHPNIQGAKSSSDVKSYIDKD----GDTLEWGEF-QVDGR-SARGGQ--------QTANDTAAKAL-NSGSAEAALAIIREELPKDFIFQ
CIGMV [DQ641692] LHVLMQFEGKFQCKNQRFFDL-VSPTRSAHFHPNIQGAKSSSDVKSYMDKD----GDTLEWGEF-QIDGR-SARGGP--------QTANDAYAQAL-NTGSKHEALKVIKELAPKDFILQ
TLCYV [AJ512761] LHVLIQFEGKYVCTNNRFFDL-VSPTRSAHFHPNIQGAKSSSDVKSYIDKD----GDTLEWGEF-QIDGR-SARGGQ--------QTANDAYAQAL-NSGSKSEALNVIKELAPKDFVLQ
HYVV [AB236325] LHVLIQFEGKYQCTNQRFFDL-VSPSRSAHFHPNIQGAKSSSDVKAYMEKD----GDVIDFGVF-QVDGR-SARGGC--------QSANDAYAEAI-NSGSKSSALSILREKAPKDFVLQ
TYLCCV [AF311734] LHVLIQFEGKFQCKNQRFFDL-TSPHRSAHFHPNIQGAKSSTDVKSYMEKD----GDVLDHGVF-QIDGR-SARGGC--------QSANDAYAEAI-NSGSKASALNILREKAPKDFVLQ
TLCNDV [U15015] IHVLIQFEGKFQCKNNRFFDL-VSPSRSAHFHPNIQGAKSASDVKNYIAKD----GDVLEWGVF-QIDGR-SARGGQ--------QTANDAYAQAI-NTGNKDDALRVLKELAPKDYVLQ
CLCRV [AM501481] LHVLIQFEGKFKCQNMRFFDL-VSPSRSAHFHPNIQGAKSSSDVKSYIEKD----GDILDWGQF-QIDGR-SARGGQ--------QTANDAYAAAL-NAGSKSEALRVIKELAPKDFVLQ
TLCSV [AY044137] LHALVQFEGKYQCTNNRFFDL-ASPSRSTHFHPNIQGAKSSSDVKSYIDKD----GDTIDWGEF-QIDGR-SARGGQ--------QSANDAYAAAL-NAGSKSEALRIIKELAPKDYILQ
TYLCSV [X61153] LHILIQFEGKYNCTNQRFFDL-VSPTRSAHFHPNIQGAKSSSDVKSYIDKD----GDVLEWGTF-QIDGR-SARGGQ--------QTANDAYAKAI-NAGSKSQALDVIKELAPRDYVLH
ACMV [J02057] LHALIQFEGKITITNNRLFDC-VHPSCSTSFHPNIQGAKSSSDVKSYLDKD----GDTVEWGQF-QIDGR-SARGGQ--------QSANDAYAKAL-NSGSKSEALNVIRELVPKDFVLQ
CoGMV [EU636712] LHALVQFQKKFVCTNCRLFDL-SHPQNSRQFHCHIETARSSSDAKSYIEKD----GVFCEWGTF-QVDGR-SARGGQ--------QTINEAYAKAL-NSGSKDEALTVIKELVPKDYVCQ
TSLCV [AF130415] LHCLIQLSGKSNIKDARFFDL-THPRRSANFHPNAQAAKDTNAVQNYITKE----GDYCESGQY-KVSG-----GTK--------SNKDDVYHNAV-NAASAGEALDIIRAGDPKTFIVS
SpSCTAV [GU734126] LHCLIQLSGKSNIKDCRFFDI-THPRRSANYHPNVQAAKDANAVHNYITKE----GDYCESGQY-KVSG-----SSK--------ANKDDVYHNAV-NSGGVKEALEIIKAGDPKTFVIQ
HrCTV [U49907] LHCLIQLSGKSNIKDCRFFDI-THPQRSTSFHPNVQSAKDTNAVKNYITKE----GDYCESGQY-KVSG-----GTK--------ANKDAVYHNAV-NAGGVREALDIIKAGDPKTFVTQ
ECSV [FJ665632] LHAIVCTSERRDIRDPRIFDF-------GEFHPKIETCRSVSKSLKYIQKE---AGSFYEHGTV-PCDKRLTGRKRK--------AEQDEWWHQAV-NSGSIEEALQLVKDNEPRTFWLQ
GRLaV-WA [JQ000000] LHALLIFKKRQAIRNPKHFDI---TMSSHSYHPNITKPRDVADVLNYVGKE----GDTVEHGTK-P-----TLKSKK--------QSRELDFGEFLRVANSKDEFLQLVKQHYPYQYVLN
GCFaV-NY [JQ901105] LHALLIFKKRQAIRNPKHFDI---TISSHSYHPNITKPRDVADVLNYVGKE----GDTVEHGTK-P-----TLKSKK--------QSRELDFGEFLRVANSKDEFLQLVKQHYPYQYVLN
BCTIV [JQ707938] THCIIQCDKKLDVNGNLFFNI-ILPD-GRTIHPRIDGLNAPKRAFEYITKE---DTSPRTFGEL-RLGGRSPNSIGN----------SNVEWRRILDSSNTKEEFFSNIRESCPTDFVLR
BCTIV [JQ707939] THCIIQCDKKLDVNGNLFFNI-ILPD-GRTIHPRIDGLNAPKRAFEYITKE---DTSPRTFGEL-RLGGRSPNSIGN----------SNVEWRRILDSSNTKEEFFSNIRESCPTDFVLR
BCTIV [EU273816] THCIIQCDKKLDVNGNLFFNI-ILPD-GRTIHPRIDGLNAPKRAFEYITKE---DTSPRTFGEL-RLGGRSPNSIGN----------SNVEWRRILDSSNTKEEFFSNIRESCPTDFVLR
BCTIV [JQ707945] THCIIQCDKKLDVNGNLFFNI-ILPD-GRTIHPRIDGLNAPKRAFEYITKE---DTSPRTFGEL-RLGGRSPNSIGN----------SNVEWRRILDSSNTKEEFFSNIRESCPTDFVLR
SCTAV [HQ443515] THCIFQCNKKLQVNGNLFFNI-ILPD-GRTIHPRIDGLNAPKRAWEYITKE---DQQPRCFGEL-RLAGRSPNRLGD----------SNLEWRRILDSSNTKDEFFNNIRESCPTDFVLR
TbYDV [M81103] LHCLIQLDKRSNIRDPSFFDL-------EGNHPNIQPAKNSEQVLEYISKD----GNVITKGEF-----K-KHRVSP--------SKSDERWRTIIQTATSKEEYLDMIKDEFPHEWATK
CpYV [JN989439] LHCLLQLDKRSNIRDPSFFDF-------EGNHPNIQPAKNSEQVLDYISKD----ENVITKGEF-R-----KHKVSP--------TKTDERWRNIINTATSKEEYLGMIKEEFPHEWATK
CpRV [GU256532] LHCLIQLTNKPNISDASFFDF-------EGNHPNIQPARNSEQVLDYISKD----GNIITKGEF-K-----KHRVSP--------TKHDERWRTIINTATSKEHYLGMIRDQFPHEWASK
CpCDV [AM850136] YHALVQLDKKPWIRDPSFFDF-------EGNHPNIQPARNSKQVLEYISKD----GDIKTRGDF-----R-DHKVSP--------RKSDARWRTIIQTATSKEEYLDMIKEEFPHEWTTK
ODV [AM296025] LHVLVQCEKRASITNPNALNLRMDLSPFTTYHPNIQPANNCNDVREYITKEVDSHEHTAEWGTF-IH----HTTSGR--------PDKDEAMKQIIESATSKEEFISMVRSRFPFEWSIN
WDV [X02869] LHVLVQNKLRASITNPNALNLRMDTSPFSIFHPNIQAAKDCNQVRDYITKEVDSDVNTAEWGTF-VA----VSTPGR--------KDRDADMKQIIESSSSREEFLSMVCNRFPFEWSIR
BCSMV [HQ113104] LHCLVQCKKYVRTKSAKFFDV-------EEFHPNVQNARMPHKVLAYIKKN---PLCFVETGVF-QASTK-QKKKKVDAP-----STKDAKMAEIIKSSTCKEDYLSMVRNTFPFDWATR
PSMV [JF905486] LHCIIQCSKYVRTTSAKFFDI-------KEFHPNVQNPRMPKKALSYCKKS---PISEAEYGVF-QEIKR-PRKKKADAP-----STKDAKMAEIIKSSTNKEDYLSMVRKSFPFDWATR
DDSMV [HM122238] LHAFLQFDKVFRTTSAKYFDF-------FEFHPNIQAARNPEKTLEYCQKN---PADFYEDGVFVKPKASRKRKLAS--------FTRDKKMKQIMANATSRDEYLSMIRKAFPFDWAIR
CSMV [M20021] LHAFVQLEANFRTTSPKYFDL-------DEFHPNIQAARQPASTLKYCMKH---PESSWEFGKF-LKPKV-NRSPTQS-------ASRDKTMKQIMANATSRDEYLSMVRKSFPFEWAVR
MiSV [D01030] LHVLIQLNTKLCTRNPKFFDV-------QGFHPNIQPVRDAEKVFGYISKT---NGDSDEMGEL-QLRIKKPEK-----------PTRDQRMAMIIASSTNRNEYLSMVRKEFPFDWAIR
EMSV [JF508490] LHALVQLEKKLFTRRQTFFDQ---FLHGTKFHPNIQPARDASKVLGYITKQ---NGEEYIFGKP-TLPKK--KKTAQ--------EGRDQRMRAIIESSTSKQEYLSMVRKEFPFDWATR
MRSV [JQ624880] LHVLLQTMRPMSTRDPSFFDI-------QGYHPNIQASRSPNKTREYILKS---PITVYSRGTF-IPRAGTSGAGYGSTP----VPKRNEIMRGIIETTTNKAEYLSEVQKAFPFEWATK
DSV [M23022] SHALVQTEKQVNTTNQRFFDI-------LEFHPNIQSAKSVNKVRTYILKN---PVEKFERGTF-VPRKS-PFLGESSSS--EKKHNKDDVMRDIIDHATSKEEYLSMVQKALPYDWATK
MSV [AF329881] LHALLQTEKPVRISDSRFFDI-------NGFHPNIQSAKSVNRVRDYILKE---PLAVFERGTF-IPRKS-PFLGKSDSEVKEKKPSKDEIMRDIISHATSKEEYLSMIQKELPFDWSTK
PanSV [L39638] CHALLQCIKPVTTRDERYFDI-------DRYHPNIQSAKSTDKVREYILKD---PKDKWEKGTY-IPRKKSFVPPGK--ENSEKKPSKDEVMKEIMTHATSRAEYLSLVQTSLPYDWATK
SacSV [GQ273988] CHALAQSVRPVTTSDPRFFDV-------NEYHPNIQSAKSVDRVREYILKD---PLCQWEKGTF-VPRKK-PFVPQIGES-SNTRASKDDIVRDIIQHSTNKHEYLSMLQKELPYEWATK
USV [EU445697] CHALAQSVKPVTTRNSRFFDI-------EDHHPNIQSAKSVDKVRAYILKD---PIALWERGTF-IPRKK-SFVPHQGDE-HTPKPTKDDIVRDIIEHSTSKQEYLSRLQNELPYEWATK
SSV [M82918] IHVLAQSAKPVYTTDSGFFDI-------DGFHPNIQSAKSANKVRAYAMKN---PVTYWERGTF-IPRKT-SFLGDSTEP-NSKKQSKDDIVRDIIEHSTNKQEYLSMIQKALPYEWATK
SSRV [AF072672] LHALVQSVRPVQTTNQGFFDI-------ESFHPNIQSAKSANKVREYILKN---PIAKWEKGTF-IPRKQ-CFVSSSSES-KNSKPSKDDIVRDIIEHSTSKEEYLSMLQKALPYDWATK
ESV [EU244915] LHALAQSVKPVYTHDERFFDI-------EDYHPNIQAAKSANKVRDYVLKN---PLKVWERGTF-IPRKK-TFLGSTSEG-NTTKQSKDDIVRDIIEHSTSKQEYLSMIQKALPYEWATK
SSEV [AF239159] IHALAQSVKPVQTTNPRFFDI-------EDFHPNIQSAKSADRVKEYVLKN---PIKQWEKGTF-IPRKK-SFATTSSED-RQPKPTKDDIVRDIIEHSTSKQEYLSMIQKALPYEWATK
Curtovirus
Turncurtovirus
Topocuvirus
Begomovirus
Eragrosvirus
Graingemvirus
Becurtovirus
Mastrevirus

```
                     RBR motif

                     ....|....|....|....|....|....|....|....|....|....|....|....|....|....|....|....|....|....|....|....|....|....|....|....|
                         245       255       265       275       285       295       305       315       325       335       345       355
BCTV-G [EU586260]    YHNLKPNLEAIFLPPPDIYQPPFPQS-SFTRVPEIVQEWADSYF-----GVDAAARP------------FRYNSLILEGDSRTGKTMWARSLGS-HNY---------ISG----------
BCTV-G [EU193175]    YHNLKPNLEAIFLPPPDIYQPPFPQS-SFTRVPEIVQEWADSYF-----GVDAAARP------------FRYNSLILEGDSRTGKTMWARSLGP-HNY---------ISG----------
BCTV-G [HQ634913]    YHNLKPNLEAIFLPPPDIYQPPFPQS-SFTRVPEIVQEWADAYF-----GVDAAARP------------FRYNSLILEGDSRTGKTMWARSLGP-HNY---------ISG----------
BCTV-G [HQ214016]    YHNLKPNLEAIFLPPPDIYQPPFPQS-SFTRVPEIVQEWADSYF-----GVDAAARP------------FRYNSLILEGDSRTGKTMWARSLGP-HNY---------ISG----------
BCTV-G [EU586261]    YHNLKPNLEAIFLPPPDIYQPPFPQS-SFTRVPEIVQEWADSYF-----GVDAAARP------------FRYNSLILQGDSRTGKTMWARSLGP-HNY---------ISG----------
BCTV-F [AY134867]    YHNLKPNLEAIFLPPPDIYQPPFPQS-SFTQVPEIVQEWADSYF-----GVDAAARP------------FRYNSIIIEGDSRTGKTMWARSLGP-HNY---------ISG----------
BCTV-F [U56975]      YHNLKPNLEAIFLPPPDIYQPPFPQS-SFTQVPEIVQEWADSYF-----GVDAAARP------------FRYNSIIIEGDSRTGKTLWARSLGP-HNY---------ISG----------
BCTV-E [EU921828]    YHNLKPNLEAIFLPPPDIYQPPLSLS-SFTRVPEIIQEWADSYF-----GVDAAARP------------FRYNSIIIEGDSRTGKTMWARSLGP-HNY---------ISG----------
BCTV-D [FJ545686]    YHNLKPNLEAIFLPPPDIYQPPFPLS-SFTRVPEIIQEWADSYF-----GVDSAARP------------FRYNSIIIEGDSRTGKTMWARSLGP-HNY---------ISG----------
BCTV-A [AY548948]    FHNLKPNLEAIFLPPPEIYKPPFPLS-SFTRVPEVIQEWADSYF-----GVDAAARP------------FRYNSIIIEGDSRTGKTMWARSLGP-HNY---------ISG----------
BCTV-P [EF501977]    YHNLKPNLEAIFLPPPDIYQPPFPLS-SFTRVPEIIQEWADSYF-----GLEPAARP------------YRYNSIIIEGDSRTGKTMWARCLGP-HNY---------ITG----------
BCTV-C [X97203]      YHNLKPNLEAIFLPPPDLFQPPFPLS-SFTRVPDIIQEWADSYF-----GLDPAA-P------------FRYNSIIIEGDSRTGKTMWARCLGP-HNY---------ITG----------
BCTV-C [U02311]      YHNLKPNLEAIFLPPPDLYQPPFPLS-SFTRVPEIIQEWADSYF-----GLDPAARP------------FRYNSLIIEGDSRTGKTMWARCLGP-HNY---------ITG----------
TCTV [GU456685]      FHNLKPNLAAIFNPPPVGYVPKYNHT-QFVLTDDILD-WLESNF-----FLEESNSPAGPSKYKVIPSIDRPKSIIIEGPSRTGKTLWARSLGS-HNY---------ITG----------
DoYMV [AM157413]     YHNLRSNLERIFAPPITVYTSFYKPT-DFSQVPSVMTDWAESNVVDPSWRSDPAARP------------RRPMSIVVEGATRTGKTLWARSLGA-HNY---------MCG----------
BGYMV [D00201]       NHNIRSNLERIFFKVPEPWVPPFPLS-SFVNIPVVMQDWVDDYF-----GRGSAARP------------ERPISIIVEGDSRTGKTMWARALGP-HNY---------LSG----------
MaMPRV [AY044133]    YHNIRSNLEKIFTPPTEPWVPPFQLS-TFNNVPLIMSDWVNENI------SDSAARP------------LRPISIIVEGPSRTGKTIWARSLGP-HNY---------LCG----------
BCTV-H [AF379637]    HHNLLNNAQKIFQRAPDPWTPLFPLS-SFTNVPEEMQEWADAYF-----GVDAAARP------------LRYNSIIVEGDSRTGKTMWARSLGA-HNY---------ITG----------
BCTV-H [M24597]      HHNLLNNAQKIFQRPPDPWTPLFPLS-SFTNVPEEMQEWADAYF-----GVDAAARP------------LRYNSIIVEGDSRTGKTMWARSLGA-HNY---------ITG----------
TGMV [K02029]        FHNLNSNLDRIFDKTPEPWLPPFHVS-SFTNVPDEMRQWAENYF-----GKSSAARP------------ERPISIIIEGDSRTGKTMWARSLGP-HNY---------LSG----------
TPCTV [X84735]       YHNLVSNLDRIFTPAPTPFVPPFQLS-SFTNVPEDMQEWADDYF-----GVSAAARP------------MRYKSIIIEGESRTGKTMWARSLGP-HNY---------LSG----------
SPLCV [AF104036]     FHNLVSNLDRIFSPPPSVYSSPFSSS-SFNAVPDIISDWAAENV------MDSAARP------------DRPISIVIEGPSRIGKTVWARSLGP-HNY---------LCG----------
CGMV [AF029217]      YHNIKSNLDRIFSKPLEPYSCPFRLN-SFNNVPQIMKDWASINV------VDAAARP------------DRPISIVIEGESRTGKTMWARSLGA-HNY---------LCG----------
TLCJV [AB100304]     YHNIKANLDRIFTPRAEVFVCPFLSS-SFDQVPEELECWVSENV------RDAAARP------------WRPVSIVIEGESRTGKTMWARSLGP-HNY---------LCG----------
CIGMV [DQ641692]     FHNLNSNLDRIFVQEVPIYRSPFLSS-SFNQVPEELELWVSENV------MSAAARP------------LRPKSIVLEGDSRTGKTMWARSLGP-HNY---------LCG----------
TLCYV [AJ512761]     FHNLNANLDRIFAPPLEVFGSPFLSFIIWIKFPKNLKNGAADNV------RHAGARP------------WRPISIVIEGESRTGKTMWARSLGP-HNY---------LCG----------
HYVV [AB236325]      FHNLNSNLDRIFAPPLEEFVSPFSSS-SFDQVPDELEEWAAENV------RDSAARP------------WRPMSIVIEGDSRTGKTMWARSLSPRHNY---------LCG----------
TYLCCV [AF311734]    FHNLNSNLDRIFTPPIVEYISPFSSS-SFDQVPEELDEWAVDNV------VSAAARP------------LRPVSIVIEGDSRTGKTMWARSLGP-HNY---------LCG----------
TLCNDV [U15015]      FHNLNTNLDRIFQPPSEVYVSPFSIS-SFDRVPADLVDWVSSNV------VCAAARP------------FRPISIVIEGDSRTGKTMWARCLGP-HNY---------LCG----------
CLCRV [AM501481]     FHNLNANLDRIFQEPPAPYVSPFSSS-SFDQVPEELEVWAAENV------VSAAARA------------NRPISVVIEGDSRTGKTMWARSLGP-HNY---------LCG----------
TLCSV [AY044137]     FHNLNSNLDRIFQEPPAPYISPFLSS-SFNQVPEELEVWVSENV------MSSAARP------------WRPNSIIIEGDSRTGKTMWARSLGP-HNY---------LCG----------
TYLCSV [X61153]      FHNINSNLDKVFQVPPAPYVSPFLSS-SFDQVPDELEHWVSENV------MDAAARP------------WRPVSIVIEGDSRTGKTTWARSLGP-HNY---------LCG----------
ACMV [J02057]        FHNLNSNLDRIFQEPPAPYVSPFPCS-SFDQVPVEIEEWVADNV------RDSAARP------------WRPNSIVIEGDSRTGKTIWARSLGP-HNY---------LCG----------
CoGMV [EU636712]     FHNLNANLERIFAPPVHVFEPPFPLS-SFNNVPAVINQWVNDNI------MDAAARP------------FRPISIVIEGPSRTGKTLWARSLGR-HNY---------LCG----------
TSLCV [AF130415]     YHNVKANIERLFQKAPEPWVPPFPLS-SFTNVPDEMQEWADDYF-----GRDAAARP------------ERPISIIIEGESRTGKTMWARALGP-HNY---------LSG----------
SpSCTAV [GU734126]   HHNVLANLERIFKKPQETWTPPFPLS-SFNNVPDEMQEWADNYF-----GQPIESRP------------IRPKSLIIEGDSRTGKTCWARSLGK-HNY---------LSG----------
HrCTV [U49907]       HHNVKANLEHIFAKPPEPWNPPYPLS-SFTNVPEEMQEWADDYF-----GRPVADS-------------HKAKSIIIEGNSRTGKTCWARALGT-HNY---------LCG----------
ECSV [FJ665632]      HHNLVTNARRIWSEVRAEFVPKYSES-SFS-VPRVLSDWVANNL--------RADPLP------------DRPLSLIIEGDSRTGKTAWARSLGR-HNY---------LSG----------
GRLaV-WA [JQ000000]  LQRLEYFAAQTWPPTPTPHANQWT---AWNNLPPEIHLWVQNEL-------------------------YV----------VRKSSAQRNLGP---G---------INN----------
GCFaV-NY [JQ901105]  LQRLEYFVAQTWPPTPTPHANQWT---AWNNLPPEIHLWVQNEL-------------------------YV--------VR--KSSAQRNLGP---G---------INN----------
BCTIV [JQ707938]     WPSILSFANYHFRPVVQPYTPRWT---EFSRLPDTIKEWAEQNI-------------------------YF-------------------VSS---D----------CL----------
BCTIV [JQ707939]     WPSILSFANYHFRPVVQPYTPRWT---EFSRLPDTIKEWAEQNI-------------------------YF-------------------VSS---D----------CL----------
BCTIV [EU273816]     WPSILSFANYHFRPVVQPYTPRWT---EFSRLPDTIKEWAEQNI-------------------------YF-------------------VSS---D----------CL----------
BCTIV [JQ707945]     WLSILSFANYHFRPVVQPYTPRWT---EFSRLPDTIKEWAEQNI-------------------------YF-------------------VSS---D----------CL----------
SCTAV [HQ443515]     WPSILAFANYHFRPVVQPYLPRWT---EFTRLPDQIKEWAEHNI-------------------------YF-------------------VSS---H----------CL----------
TbYDV [M81103]       LQWLEYSANKLFPPQPEIYQATFTE--EDLQCHEDLQLWRDQHL-------------------------YH-------------------VSV---D----------A------------
CpYV [JN989439]      LQWLEYSANKLFPEIEPPYQSPFTS--CSLQCHEKITDWLNRDL-------------------------YL-------------------VSL---D-----------------------
CpRV [GU256532]      LQWFEYSANKLFPDVEPPYQSPFPE--ASLQCHEEIQDWLNRDL-------------------------YL-------------------VSV---E----------A------------
CpCDV [AM850136]     LQWLEYSANKLFPPQPEAYVSPFTE--SDLRCHEDLAQWRDTHL-------------------------YQ-------------------VSI---D----------A------------
ODV [AM296025]       LQRFQYTANYLFPDPIPQYTPEFPT--ESLICHETIQNWANTEL-------------------------FT-------------------VRH---R------SLYICGPTRTGKTSWA
WDV [X02869]         LKDFEYTARHLFPDPVATYTPEFPT--ESLICHETIESWKNEHL-------------------------YS-------------------VSL---E-----------------------
BCSMV [HQ113104]     LQQFQYSAESLFPSVPTPYMDPFGM--PAQDEHPVIGAWLQAEL-------------------------FS-------------------VSP---Y-----------------------
PSMV [JF905486]      LQQFQFSAESLFPSTPPPYVDPFGM--PSQDTHPVIGAWLRDEL-------------------------YT-------------------VSP---Y-----------------------
DDSMV [HM122238]     LQQFEYSAKALFPEAPIQYQPQFVSN-DMSDHP-VIGEWLDTEF-------------------------FT-------------------VSV---S-----------------------
CSMV [M20021]        LQQFQYSANALFPDPPQTYSAPYASR-DMSDHP-VIGEWLQQEL-------------------------YT-------------------VSP---Q-----------------------
MiSV [D01030]        LQQFEYSAAALFTEPPPVYQSPFPN--EQIVCPPELVDIIDQEW-------------------------NQ-------------------VTT---D-----------------------
EMSV [JF508490]      LMQFEYSASSLFPEPPVEYTSPFPV--DQLLCPEDITEIINSEW-------------------------FQ-------------------VTP---Y-----------------------
MRSV [JQ624880]      LQQFEYSAERLFPTLPSPFVPPHPPSEPDLHCYETIRSWKDENI-------------------------FQ-------------------VHP---Y----------I------------
DSV [M23022]         LSYFEYSADRLFPVEAAPFINPHPPSEPDLLCQETITDWLQNDL-------------------------FQ-------------------VSA---E----------A------------
MSV [AF329881]       LQYFEYSANKLFPEIQEEFTNPHPPSSPDLLCNESINDWLQPNI-------------------------FQ-------------------VSP---E----------A------------
PanSV [L39638]       LSYFEYSASRLFPDIAEPYSNPHPATDPDLLCNETLQDWLEPNI-------------------------YQ-------------------VSP---K----------A------------
SacSV [GQ273988]     LQYFEYSANKLFPEIAEPYTNPHPPTQPDLHCYERIEEWLNFNV-------------------------YQ-------------------VQP---QEAGRARSLYIVGPTRTGKSTWA
USV [EU445697]       LQYFEYSANKLFPDIPEPYIHPHPQTEPELHCKETIDDWLKPNI-------------------------FQ-------------------VHP---D-----------------------
SSV [M82918]         LQYFEYSANKLFPDIQEIYTSPFPQSTPALLDPTAINTWLENNL-------------------------YQ-------------------VSP---S----------A------------
SSRV [AF072672]      LQYFEYSASKLFPDTVEEYTSPHPTTTPLLRDPTTIDNWVQPNL-------------------------FQ-------------------VSP---E----------A------------
ESV [EU244915]       LQYFEYSANRLFPEIQETYTNPHPPTAPQLQDGETIQSWVYTNI-------------------------YQ-------------------VSP---Q----------A------------
SSEV [AF239159]      LQYFEYSASRLFPETAEVYTNPHPPTEPDLINFETIEDWLNPNI-------------------------YQ-------------------VSP---E----------A------------
```

Curtovirus

Turncurtovirus

Topocuvirus

Begomovirus

Eragrosvirus

Graingemvirus

Becurtovirus

Mastrevirus

```
                         ....|....|....|....|....|....|....|....|....|....|....|....|....|....|....|....|....|....|....|....|....|....|....|....|
                            365        375        385        395        405        415        425        435        445        455        465        475
BCTV-G [EU586260]        ---------HLDFSLKTYHDDVLYNVIDDVDPSYLKMKHWKHLIGAQREWQTNLKYG-KPRVIKGGI-PSI---ILCNPGDGSSYQDFLDKSENEALRSW-TIQNSVFIKISSPLFDKDT
BCTV-G [EU193175]        ---------HLDFSLKTYHDDVLYNVIDDVDPSYLKMKHWKHLIGAQREWQTNLKYG-KPRVIKGGI-PSI---ILCNPGDGSSYQDFLDKSENEALRSW-TIQNSIFFKISSPLFDKDT
BCTV-G [HQ634913]        ---------HLDFSLKTYHDDVLYNVIDDVDPSYLKMKHWKHLIGAQREWQTNLKYG-KPRVIKGSI-HSI---ILCNPGDGSSYQDFIDKSENEALTSW-TIQNSVFIKISSPLFDKDT
BCTV-G [HQ214016]        ---------HLDFSLKTYHDDVSYNVIDDVDPSYLKMKHWKHLIGAQREWQTNLKYG-KPRVIKGGI-PSI---ILCNPGDGSSYQDFLDKSENEALRSW-TIQNSIFFKISSPLFDKDT
BCTV-G [EU586261]        ---------HLDFSLKTYHDDVLYNVIDDVDPSYLKVKHWKHLIGAQREWQTNLKYG-KPRVIKGGI-PSI---ILCNPGDGSSYEDFLDKSENEALXSW-TIQNSIFFKISSPLFDKDT
BCTV-F [AY134867]        ---------HLDFSLKTYHDDVLYNVIDDVDPAYLKMKHWKHLIGAQREWQTNLKYG-KPRVIKGGI-PSI---ILCNPGDGSSYQDFLDKSENEALKSW-TIQNSAFVKLSGPLFDKDT
BCTV-F [U56975]          ---------HLDFSLKTYHDDVLYNVIDDVDPAYLKMKHWKHLIGAQREWQTNLKYG-KPRVIKGGI-PSI---ILCNPGDGSSYQDFLDKSENEALKSW-TIQNSTFVKLSGPLFDKDT
BCTV-E [EU921828]        ---------HLDFSLKTYNDDVLYNVIDDVDPNHLKMKHWKHLIGAQREWQTNLKYG-KPRVIKGGI-PSI---LLCNPGEGSSYQDFLDKSENEALKSW-TLQNSIFAKLSGPLFDKDT
BCTV-D [FJ545686]        ---------HLDFSLKTYNDDVLYNVIDDVDPNYLKMKHWKHLIGAQREWQTNLKYG-KPRVIKGGI-PSI---ILCNPGEGSSYQDFLDKSENEALRSW-TLQNSVFAKLTGPLFDNNQ
BCTV-A [AY548948]        ---------HLDFSLKTYSDDVLYNVIDDVDPNYLKMKHWKHLIGAQREWQTNLKYG-KPRVIKGGI-PSI---ILCNPGEGSSYQDFLDKQENEALKSW-TLQNSTFAKIWGPLFDKAS
BCTV-P [EF501977]        ---------HLDFSLKTYSDNVLYNVIDDVDPNYLKMKHWKHLIGAQREWQTNLKYG-KPRVIKGGI-PSI---ILCNPGEGSSYQDFLNKSENEALKSW-TLQNSVFAKLTSPLFDNNQ
BCTV-C [X97203]          ---------HLDFSLKTYSDNVLYNVIDDVDPNYLKMKHWKHLIGAQREWQTNLKYG-KPRVIKGGI-PSI---ILCNPGEGSSYQDFLNKSENEALRSW-TLQNSVFAKLTSPLFDNNQ
BCTV-C [U02311]          ---------HLDFSLKTYNDNVLYNVIDDVDPNYLKMKHWKHLIGAQREWQTNLKYG-KPRVIKGGI-PSI---ILCNPGEGSSYQDFLNKSENEALRSW-TLQNSVFAKLTSPLFDNNQ
TCTV [GU456685]          ---------HLDFSTKVYNDDVSYNVIDDVDPHYLKMKHWKHLIGAQKEWQTNLKYG-KPRIIKGGI-PSI---ILCNPGDGASYQNFLDKPENEALKSW-SLQNSVFTTIEEPLFNITS
DoYMV [AM157413]         ---------HLDLSPKIFSNDAWYNVIDDVDPHY--LKHFKEFMGAQMDWQSNIKYG-KPTQIKGGI-PTI---FLCNPGPRSSYKEFLDEEQNESLKEW-AYKNAVFVTLEQPLFSTEH
BGYMV [D00201]           ---------HLDFNSRVYSNSVEYNVIDDISPNYLKLKHWKELIGAQKDWQSNCKYG-KPVQIKGGI-PSI---FLCNPGEGSSYKDFLNKEENRALHNW-TIHNAIFVTLTAPLYQSTA
MaMPRV [AY044133]        ---------HIDLNPRIYSNHAWYNVIDDVDPHY--LKHFKEFMGAQRDWQSNCKYG-KPIQIKGGI-PTI---FLCNPGPHSSYKEYLSEEKNKSLNDW-AQKNAIYVSIEEPLYSTFN
BCTV-H [AF379637]        ---------HLDFSLRTYYDEVEYNVIDDVDPTYLKMKHWKHLIGAQKEWQTNLKYG-KPRVIKGGI-PCI---ILCNPGPESSYQQFLEKPENEALKSW-TLHNSTFCKLQGPLFNNQA
BCTV-H [M24597]          ---------HLDFSPRTYYDEVEYNVIDDVDPTYLKMKHWKHLIGAQKEWQTNLKYG-KPRVIKGGI-PCI---ILCNPGPESSYQQFLEKPENEALKSW-TLHNSTFCKLQGPLFNNQA
TGMV [K02029]            ---------HLDLNSRVYSNKVEYNVIDDVTPQYLKLKHWKELIGAQKDWQTNCKYG-KPVQIKGGI-PSI---VLCNPGEGASYKVFLDKEENTPLKNW-TFHNAKFVFLNSPLYQSST
TPCTV [X84735]           ---------HLDFNSRVYSNSALYNVIDDVTPHYLKLKHWKELIGAQRDWQSNCKYG-KPVQIKGGI-PSI---VLCNPGGDTSFQDFLDKEENEALKDW-TLYNAVFIKLTEPLYDGTV
SPLCV [AF104036]         ---------HLDLSPKVYSNSAWYNVIDDVNPQY--LKHFKEFMGAQKDWQSNCKYG-KPVRIKGGI-PTI---FLCNPGEGSSFKLWLDKPEQGALKNW-ATANAIFCDVQSPFWVQEE
CGMV [AF029217]          ---------HLDLSPKVYSNNAWYNVIDDVDPHY--LKHFKEFMGAQHDWQSNTKYG-KPIQIKGGI-PTI---FLCNPGPNSSYKSFLDEDKNKDLKQW-ALKNAIFVDISFPLYSNSN
TLCJV [AB100304]         ---------HLDLSPKVYSNDAWYNVIDDVDPHY--LKHFKEFMGAQRDWQSNTKYG-KPIQIKGGI-PTI---FLCNPGPTSSYKEYLDEEKNSALKEW-AIKNAEFVTLNSPLYSGTN
CIGMV [DQ641692]         ---------HLDLSPKVYSNDAWYNVIDDVDPHY--LKHFKEFMGAQRDWQSNTKYG-KPVQIKGGI-PTI---FLCNPGPGASYIQFLNEEKQSALKTW-ALKNAEFVTLTEPLYSSTN
TLCYV [AJ512761]         ---------HLDLSPKVYSNDAWYNVIDDVDPHY--LKHFKEFMGAQRDWQSNTKYG-KPIQIKGGI-PTI---FLCNPGPTSSYKEFLEEEKNSALKEW-ALKNAEFITLNSPLYSGTN
HYVV [AB236325]          ---------HLDLSPKVYSNEAWYNVIDDVDPHY--LKHFKEFMGAQRDWQSNTKYG-KPIQIKGGI-PTI---FLCNPGPTSSYTEYLDEEKNAALKAW-AIKNATFVTLYGPLYSGTH
TYLCCV [AF311734]        ---------HLDLSPKVYSNDAWFNVIDDVDPHY--LKHFKEFMGAQRDWQSNTKYG-KPVQIKGGI-PTI---FLCNPGPNSSYKEYLEEEKNSALRNW-AVKNAIFVSLNGPLYSGSY
TLCNDV [U15015]          ---------HLDLSPKVYSNDAWYNVIDDVDPHY--LKHFKEFMGAQRDCQSNTKYG-KPVMIKGGI-PTI---FLCNKGPNSSYKEYLDEEKNAALKQW-AIKNAVFITLEEPLYSGRE
CLCRV [AM501481]         ---------HLDLSPKVYSNDAWFNVIDDVDPHY--LKHFKEFMGAQKDWQSNTKYG-KPVQIKGGI-PTI---FLCNPGPNSSYKEFLDEEKNSALKNW-ALKNAIFITLDRPMYSGTN
TLCSV [AY044137]         ---------HLDLSPKVYSNDAWYNVIDDVDPHY--LKHFKEFMGAQRDWQSNTKYG-KPIQIKGGI-PTI---FLCNPGPTSSYKEYLDEEKNISLKNW-ALKNATFVTLYEPLFSSTN
TYLCSV [X61153]          ---------HLDLSQKVYSNNAWYNVIDDVDPHY--LKHFKEFMGAQRDWQSNTKYG-KPIQIKGGI-PTI---FLCNPGPQSSFKEYLDEEKNQALKNW-ATKNAIFVTIHQPLFADTN
ACMV [J02057]            ---------HLDLSPKVFNNAAWYNVIDDVDPHY--LKHFKEFMGSQRDWQSNTKYG-KPVQIKGGI-PTI---FLCNPGPTSSYKEFLAEEKQEALKAW-ALKNAIFITLTEPLYSGSN
CoGMV [EU636712]         ---------HLDLSPKVYSNEAWYNVIDDVDPHY--LKHMKEFMGAQRDWQSNCKYG-KPIQINGGI-PTI---FLCNPGPTSSYKEYFEEEKNSAIYDW-AKKNAIFVTIEEPFFDTAD
TSLCV [AF130415]         ---------HLDFNSRVYSNEAEYNVIDDITPQYLKMKHWKELIGAQKDWQSNCKYG-KPVQIKGGI-PSI---VLCNPGEGASYKSFLEKEENKALKDW-TLHNAKFIFLNSTLYQA--
SpSCTAV [GU734126]       ---------HLDFNPRVYSNEVEYNVIDDIAPSYLKLKHWKELIGAQHDWQSNCKYG-KPVQIKGGI-PSI---ILCNPGEGSSFKDFLDKDENESLKRW-TLYNAVFISISEPLYNKDD
HrCTV [U49907]           ---------HLDFNMRVYSNEVEYNVIDDVAPTYLKMKHWKELIGAQRDWQSNCKYG-KPVQIRGGV-PTI---ILCNPGEGSSFRDFLDKPENDGLRQW-TLYNAIFIFLDTPLFDNGI
ECSV [FJ665632]          ---------HLDLNGAVFDNEASYNVIDDVNPKY--LKHWKEFIGAQKDWQSNLKYG-KPVLVKGGK-PAI---VLCNS--DQSYKSFLDCEENHQLRSW-TSKNALFVDIQDALFGGVS
GRLaV-WA [JQ000000]      ---------FLEQETK----RVQHIIDDKLDAYYLDY--------VERIYNTDDELN------NAGS----------------------DAPKEEEPGD---------------------
GCFaV-NY [JQ901105]      ---------FLEQETK----RVQHIIDDKLDAYYLDY--------VERIYNTDDELN------NAGS----------------------DAPKEEEPGD---------------------
BCTIV [JQ707938]         ---------HYEICENCRGA-----ILQDRQ---MTLEEYFHLDRLSRDL--ACSED-TNVLNLSRS-QTT---DQSDQ-EVSTYADHPEQERPSGQDL---------------------
BCTIV [JQ707939]         ---------HYEICENCRGA-----ILQDRQ---MTLEEYFHLDRLSRDLACSEDTN-VLNLSRSQT-------TDQSDQEVSTYADHPEQERPSGQDL---------------------
BCTIV [EU273816]         ---------NYEICQNCRGA-----ILQDIE---MTLEEYFHLDRLSRDL--ACSED-TNVINFSRS-QTT---DQSDQ-EVSTYADHPEQERPSGQDL---------------------
BCTIV [JQ707945]         ---------HYEICDNCRGA-----ILQDRQ---MTLEEYFHLDRLSRDLTCSEDTN-VLNLSRSQT-------TDQSDQEVSTYADHPEQERPSGQDL---------------------
SCTAV [HQ443515]         ---------NYELCNNCRGS-------------------------ILQDWEISIAEH-YHIERLGDE-------LTCSENPND-----LNSSRNQTTDQS-DPEVSTSAVLQGQERPNGQ
TbYDV [M81103]           -----------------------YRLVHNVTLVE---------AHSDLVWMDDISRN-LEGLEPGSP-PST---SADQV---VPERQHGPEASEGTITGM-GPSTSLSMMTTRPTTSSTT
CpYV [JN989439]          ---------AYQL----------------VHPHLDCLSAVNDLF-----WMADYTRNQSSSDTEEEV---------------STYVDQQEQGRPVGPEAS-DCTTTSTEELTSPPTTP--
CpRV [GU256532]          ---------YTLIHPNLNTQTATEDLI----------------------WMDHYTRNPNNSDIEGS--PST---YVDQPEQERHPGQGLSGGTTTSTEEW-TSQPTTNRQHTTSSTTSHS
CpCDV [AM850136]         ---------YTLVHPVSYQQ----------------------AQSDLEWMADLTRN------REGL-------ELESP--ASTSADQLVPERPPGLEAS-DDTTGMGPSTSPRTTNTR
ODV [AM296025]           RSLGIHNYYNSQVDFTNYNADALYNIIDDIPIKY--VPNWKCFLGAQKDFTVNPKYG-KKKTIRGGI-PCI---VLVNPDE-----DWLKDMTPLQSDYL-YANAEIHYMEDGETFINHS
WDV [X02869]             ---------SYILCTSTPADQA----------------------QSDLEWMDD-----YSRSHRGGISPST---SAGQP----------EQERLPGQGL---------------------
BCSMV [HQ113104]         ---------------------AYSIHNGVT---------EDQARLDLQWMSDISRT-D--------------VLMNEEDHSTFADQQELERLLGLEVW-ELTTTGNTASTSST-----
PSMV [JF905486]          ---------------------AYSIHNGVS---------EEQAKLDLQWMSDLSR--TEVLLNEDV-PST---YVVQQEQERLLGQEVSEVITTGNIPW-TSST---------------
DDSMV [HM122238]         ----------------AYSVHALVS----------------EEQAKLDLQWMSDVTRN-EVRIIEDD--PYI---SVDQQ----------ELERLLGPEVL-ELTTTGNTL----------
CSMV [M20021]            ---------ALSLHAGISEEQA---------------------RIDLQWMSDLT---RSGALESGD-------EACTSVGQQELERLLGPEVLELITTG-STQ----------------
MiSV [D01030]            -------------------------------------------------------------------------------------------------------------------------
EMSV [JF508490]          ---------------------SYTLIH---------------------------------------PGT---SISQAADD------LQQMQDISMEHQ-EAGHVASTSVDQPVLERRL
MRSV [JQ624880]          ---------YMLEHPHCQSVEQ---------------------AAESLKWMDECTRG-----------------MLRHA------------PQDQGAST--SLGLHARARLPGPEASTQ-
DSV [M23022]             ---------YCLLNPTCYT---------------------REEAISDLQWMSDYTRS-----------------RMGSENAASTSSVQQELESLLGPEA---------------------
MSV [AF329881]           ---------YMLLQPTCYTLED---------------------AISDLQWMDSVSSH-----------------QMKDQESRASTSSAQQEPENLLGPEA---------------------
PanSV [L39638]           ---------YMLLEPSCLSLEQ---------------------AKADLEWLSETTRLFQEQESEAST-SSAQHGQVKHPGPEASDGTTTGRITSTGLHTMKKLRTMSWTTFPSSSVRAGS
SacSV [GQ273988]         RSLGRHNYWQNNVDWSSYDEEAVLNVIDDIPFKY--CPCWKQLVGCQKNYVVNPKYGKKKKVAKRSI-PAV---ILANEDE-----DWLRDMTPAQRDYM-EANCEVYIMSSGEKWFTPA
USV [EU445697]            ---------------------AYMLINNCT---------REQAESDLVWLDDVTHS-N----------------YLQTESRASTSSGLQGQGNQPGLEA---------------------
SSV [M82918]             ---------YMLANPSCLTLEE---------------------ATSDLIWMHETS---RTLIPTGSS-ACT---SSGQQ----------EQASPPGPGAW-EDIITGRTTSTGPPTTRTQ
SSRV [AF072672]          ---------YMLCNPHCLSLEE---------------------ASSDLEWMSSTS---RTIQVQESL-AST---SSAQQ----------ELENLLGPVAW-EDIITGKTTSTGPATTRTQ
ESV [EU244915]           ---------YMLLNPDCLSLED---------------------AAFDLEWMSHTS---RIFQEQGSR-AST---SSAQQ----------EQESPLGPEAW-DDIITGRTTSTGPHTTRTQ
SSEV [AF239159]          ---------YMLLTPTCLTLEE---------------------AISDLEWMQSAS---RTSQGTGNS-LCT---SLGQQ----------EQGNQHGPEA---------------------
```

Curtovirus

Turncurtovirus

Topocuvirus

Begomovirus

Eragrosvirus

Graingemvirus

Becurtovirus

Mastrevirus

```
                        ....|....|....|....|....|...
                            485       495       505
BCTV-G [EU586260]       EASSQGQTSL------------------
BCTV-G [EU193175]       EASSQSQAAL------------------
BCTV-G [HQ634913]       EASSQGQTSL------------------
BCTV-G [HQ214016]       EASSQGQASL------------------
BCTV-G [EU586261]       EASSQGQASL------------------
BCTV-F [AY134867]       EASSQGQSPL------------------
BCTV-F [U56975]         EASSQGQSPL------------------
BCTV-E [EU921828]       ETSSQGQSPL------------------
BCTV-D [FJ545686]       EASSQDQSSL------------------
BCTV-A [AY548948]       ETPQEGHSTM------------------
BCTV-P [EF501977]       EASSQDQSSL------------------
BCTV-C [X97203]         EASSQDQTSL------------------
BCTV-C [U02311]         EASSQDQTSL------------------
TCTV [GU456685]         DQEVEDSTPTV-----------------
DoYMV [AM157413]        QGTASTGEEAQNDSA-------------
BGYMV [D00201]          QDCQT-----------------------
MaMPRV [AY044133]       QTASSSRQETDST---------------
BCTV-H [AF379637]       AASSQGDSTL------------------
BCTV-H [M24597]         AASSQGDSTL------------------
TGMV [K02029]           QSS-------------------------
TPCTV [X84735]          ----------------------------
SPLCV [AF104036]        VSHSGATAHRGEEGQEESS---------
CGMV [AF029217]         KSQTQNS---------------------
TLCJV [AB100304]        QSATQNSQEEASSQA-------------
CIGMV [DQ641692]        QGPTQDCQET------------------
TLCYV [AJ512761]        QSATQNSQEEASSQAES-----------
HYVV [AB236325]         QGATQIREEESTSPEEN-----------
TYLCCV [AF311734]       QGATPNRQEDNQTTTG------------
TLCNDV [U15015]         NIALPEEEEEHSQEAS------------
CLCRV [AM501481]        QSTAQGSEEAQQEEESRS----------
TLCSV [AY044137]        QGPTQNSQETTNQA--------------
TYLCSV [X61153]         QNTTSHRQEEASEA--------------
ACMV [J02057]           QSHSQTSQEASHPA--------------
CoGMV [EU636712]        QASTSALEESNSS---------------
TSLCV [AF130415]        ----------------------------
SpSCTAV [GU734126]      ----------------------------
HrCTV [U49907]          H---------------------------
ECSV [FJ665632]         LTMREQTREDDPESPMWASDSDPGDQAV
GRLaV-WA [JQ000000]     ----------------------------
GCFaV-NY [JQ901105]     ----------------------------
BCTIV [JQ707938]        ----------------------------
BCTIV [JQ707939]        ----------------------------
BCTIV [EU273816]        ----------------------------
BCTIV [JQ707945]        ----------------------------
SCTAV [HQ443515]        DR--------------------------
TbYDV [M81103]          SPSNSSHSGSN-----------------
CpYV [JN989439]         ----------------------------
CpRV [GU256532]         SSARTGNN--------------------
CpCDV [AM850136]        PTMSLTTSPSSSSHCGSN----------
ODV [AM296025]          FTFGEGATASQ-----------------
WDV [X02869]            ----------------------------
BCSMV [HQ113104]        ----------------------------
PSMV [JF905486]         ----------------------------
DDSMV [HM122238]        ----------------------------
CSMV [M20021]           ----------------------------
MiSV [D01030]           ----------------------------
EMSV [JF508490]         GLEA------------------------
MRSV [JQ624880]         ----------------------------
DSV [M23022]            ----------------------------
MSV [AF329881]          ----------------------------
PanSV [L39638]          SSSAVKKTT-------------------
SacSV [GQ273988]        ----------------------------
USV [EU445697]          ----------------------------
SSV [M82918]            NTTSSTTYPSSTVHAGSN----------
SSRV [AF072672]         STTSLTTSPSSSVLAGSN----------
ESV [EU244915]          SSTS------------------------
SSEV [AF239159]         ----------------------------
```

Curtovirus

Turncurtovirus

Topocuvirus

Begomovirus

Eragrosvirus

Graingemvirus

Becurtovirus

Mastrevirus
