# Supplementary material for: A Leafhopper-Transmissible DNA Virus with Novel Evolutionary Lineage in the Family Geminiviridae Implicated in Grapevine Redleaf Disease by Next-Generation Sequencing
Source: PLoS One. 2013 Jun 5;8(6):e64194. doi: 10.1371/journal.pone.0064194 (PMC3673993; doi:10.1371/journal.pone.0064194)
Supplement: Figure S3 — Multiple alignment of predicted amino acid sequence of the coat protein of Grapevine redleaf-associated virus (GRLaV) and representatives of approved genera in the family Geminiviridae . Name of each genus in the family is listed on the right and corresponding amino acid sequence of individual viruses within each genus is listed on the left in the same color. Note that only the abbreviation for each virus with corresponding accession number in the parenthesis is listed. Amino acid motifs conserved (light green) and unique (yellow, orange and grey) to specific genera are highlighted. Motif in yellow was determined to be critical for leafhopper transmission of Beet mild curly top virus [57] and the motif in orange is a conserved zinc finger motif in begomoviruses [58]. The motif of unknown function is highlighted in grey color. (PDF) [file pone.0064194.s003.pdf]

Figure S3

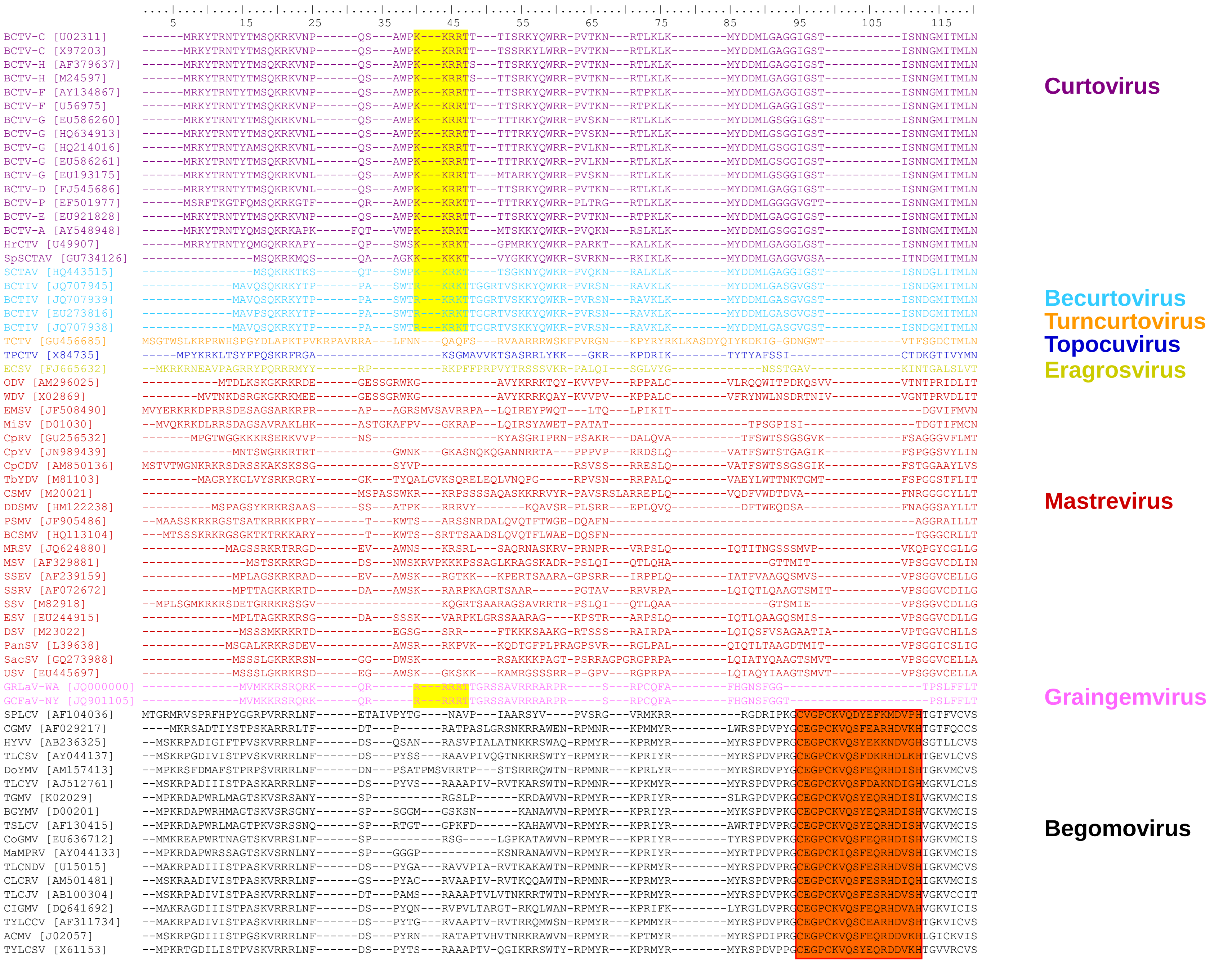

Curtovirus
Becurtovirus
Turncurtovirus
Topocuvirus
Eragrosvirus
Mastrevirus
Graingemvirus
Begomovirus

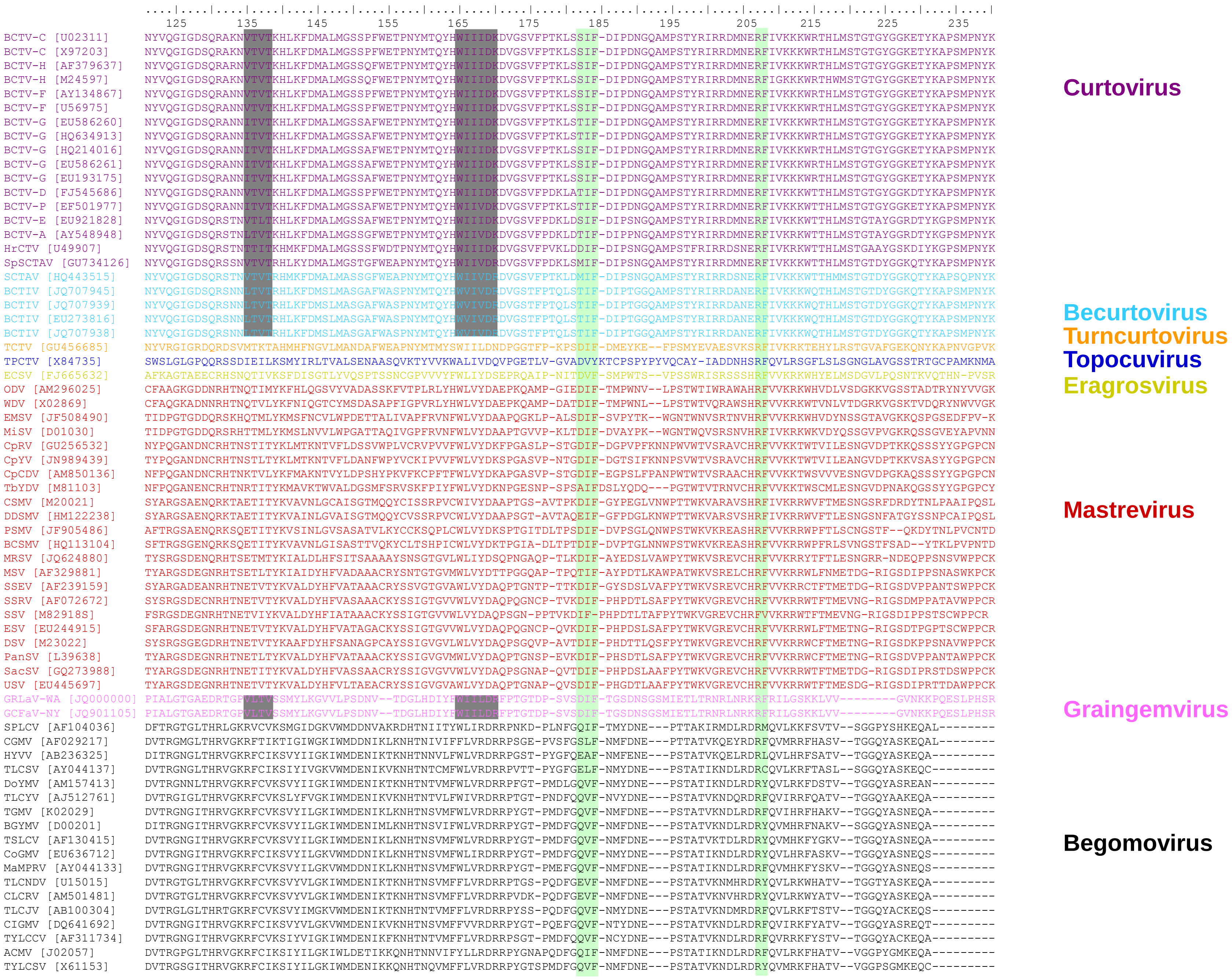

125
135
145
155
165
175
185
195
205
215
225
235
BCTV-C [U02311]
BCTV-C [X97203]
BCTV-H [AF379637]
BCTV-H [M24597]
BCTV-F [AY134867]
BCTV-F [U56975]
BCTV-G [EU586260]
BCTV-G [HQ634913]
BCTV-G [HQ214016]
BCTV-G [EU586261]
BCTV-G [EU193175]
BCTV-D [FJ545686]
BCTV-P [EF501977]
BCTV-E [EU921828]
BCTV-A [AY548948]
HrCTV [U49907]
SpSCTAV [GU734126]
SCTAV [HQ443515]
BCTIV [JQ707945]
BCTIV [JQ707939]
BCTIV [EU273816]
BCTIV [JQ707938]
TCTV [GU456685]
TPCTV [X84735]
ECSV [FJ665632]
ODV [AM296025]
WDV [X02869]
EMSV [JF508490]
MiSV [D01030]
CpRV [GU256532]
CpYV [JN989439]
CpCDV [AM850136]
TbYDV [M81103]
CSMV [M20021]
DDSMV [HM122238]
PSMV [JF905486]
BCSMV [HQ113104]
MRSV [JQ624880]
MSV [AF329881]
SSEV [AF239159]
SSRV [AF072672]
SSV [M82918S]
ESV [EU244915]
DSV [M23022]
PanSV [L39638]
SacSV [GQ273988]
USV [EU445697]
GRLaV-WA [JQ000000]
GCFaV-NY [JQ901105]
SPLCV [AF104036]
CGMV [AF029217]
HYVV [AB236325]
TLCSV [AY044137]
DoYMV [AM157413]
TLCYV [AJ512761]
TGMV [K02029]
BGYMV [D00201]
TSLCV [AF130415]
CoGMV [EU636712]
MaMPRV [AY044133]
TLCNDV [U15015]
CLCRV [AM501481]
TLCJV [AB100304]
CIGMV [DQ641692]
TYLCCV [AF311734]
ACMV [J02057]
TYLCSV [X61153]
Curtovirus
Becurtovirus
Turncurtovirus
Topocuvirus
Eragrosvirus
Mastrevirus
Graingemvirus
Begomovirus

```
                    ....|....|....|....|....|....|....|....|....|....|....|....|....|....|...
                       245       255       265       275       285       295       305
BCTV-C [U02311]     KP--MNINVRNLNMRTIWK-DTGGGKYEDVKENALLYVVVNDNT-----DNTNMYATLFGNCRCYFY------
BCTV-C [X97203]     KP--MNINVRNLNMRTIWK-DTGGGKYEDVKENALLYVVVNDNT-----DNTNMYATLFGNCRCYFY------
BCTV-H [AF379637]   KP--MNINVRNLNMRTIWK-DTGGGKYEDVKENALLYVVVNDNT-----DNTNMYATLFGNCRCYFY------
BCTV-H [M24597]     KP--MNINVRNLNMRTIWK-DTGGGKYEDVKENALLYVVVNDNT-----DNTNMYATLFGNCRCYFY------
BCTV-F [AY134867]   KP--MNINVRNLNMRTIWK-DTGGGKYEDVKENALLYVVVNDNT-----DNTNMYATLFGNCRCYFY------
BCTV-F [U56975]     KP--MNINVRNLNMRTIWK-DTGGGKYEDVKENALLYVVVNDNT-----DNTNMYATLFGNCRCYFY------
BCTV-G [EU586260]   KP--MNVNVRNLNLRTIWK-DTGGGKYEDVKENALLYVVVNDNT-----DNTNMYATLFGNCRCYFY------
BCTV-G [HQ634913]   KP--MNVNVRNLNLRTIWK-DTGGGKYEDVKENALLYVVVNDNT-----DNTNMYATLFGNCRCYFY------
BCTV-G [HQ214016]   KP--MNVNVRNLNLRTIWK-DTGGGKYEDVKENALLYVVVNDNT-----DNTNMYATLFGNCRCYFY------
BCTV-G [EU586261]   KP--MNVNVRNLNLRTIWK-DTGGGKYEDVKENALLYVVVNDNT-----DNTNMYATLFGNCRCYFY------
BCTV-G [EU193175]   KP--MNVNVRNLNLRTIWK-DTGGGKYEDVKENALLYVVVNDNT-----DNTNMYATLFGNCRCYFY------
BCTV-D [FJ545686]   KA--MNINVRNLNVRTIWK-DTGGGKYEYVKENAIIYVVVNDNT-----DNTNMYATLFGNCRCYFY------
BCTV-P [EF501977]   KP--MNMSVRNLNIKTIWK-DSGGGKYEDVKENAIIYVVVNDNS-----DNTNMYATLFGNCRCYFY------
BCTV-E [EU921828]   KP--MNINVRNLNVRTIWK-DTGGGKYEDVKENAIIYVVVNDNT-----DNTNMYATLFGNCRCYFY------
BCTV-A [AY548948]   KA--MNINIRNLNIRTVWK-DTGGGKYEDVKENAIIYVVVNDNT-----DNTNMYATLFGNCRCYFY------
HrCTV [U49907]      KG--MNINIRNLNVKTIWK-DTGGGKYEDVKENAILYIVVNDNA-----DNTNMYATLFGNCRCYFY------
SpSCTAV [GU734126]  KP--LNVNVRNINVKTVWK-DTGGGKYEDVKENAILYVVVNDNT-----DNTNMYATLYGNCRCYFY------
```

Curtovirus

```
SCTAV [HQ443515]    KA--MNINVRNINVKTIWK-DTGGGKYEDVKENALLYVVVNDNT-----DNTNMYATLFGNCRCYFY------
BCTIV [JQ707945]    KA--MNINIRNINVKTLWK-DTGGGKYEDVKENAILYVVVNDNT-----DNTNMYATLFGNCRAYFY------
BCTIV [JQ707939]    KA--MNINIRNINVKTLWK-DTGGGKYEDVKENAILYVVVNDNT-----DNTNMYATLFGNCRAYFY------
BCTIV [EU273816]    KA--MNINIRNINVKTLWK-DTGGGKYEDVKENAILYVVVNDNT-----DNTNMYATLFGNCRAYFY------
BCTIV [JQ707938]    KA--MNINIRNINVKTLWK-DTGGGKYEDVKENAILYVVVNDNT-----DNTNMYATLFGNCRAYFY------
```

Becurtovirus

```
TCTV [GU456685]     KP--IRMKFRNIWQPSEWK-DTAGGKYEDLKKGALLYVCICDNK------ATQFSFNLKGQWTMYFINRDLMY
```

Turncurtovirus

```
TPCTV [X84735]      S---INKFCKNLNVRCVYDADSATGDIASIKRGAVYLVIWPD-----------VEIRYGFSCTMYHRN-GNA-
```

Topocuvirus

```
ECSV [FJ665632]     NMMDFSKYINNLGVPTEWM-STGDGTIGDIKKGALYLAAACRQGIVGDATKITIEVEFIGQSRTYFKSIGYQ-
```

Eragrosvirus

```
ODV [AM296025]      NIVDCNKFIKGLRVSTEWM-NTGDGKIGDIKKGALYLVTCTRAGVTGDSASTGFSVVCNLTHACYFKSIGLQ-
WDV [X02869]        NIVDANKFFKGLRVTTEWM-NTGDGKIGDIKKGALYLISSTRGGVTGDSASTAFDVVCAYTHACYFKAIGIQ-
EMSV [JF508490]     NVVECNKFFEKLRVKTEWL-NTTDGTIGSVKKGALYLVANTRQMPAGDAVTTTCTTYMQGSTRLYFKSLGYQ-
MiSV [D01030]       VVE-CNKFFEKLRVKTEWA-NTSTGAIGDVKKGALYLCANTRQMPAGDSVTTSCTTMMQGSTRLYFKVLGNQ-
CpRV [GU256532]     QWRVSNKFFKRLGVSTEWK-NSSTGDVGDIKEGALYIVVAPSQS---------CDVYVNGYFRVYFKSVGNQ-
CpYV [JN989439]     QWKSSNKFFKRLGVSTEWK-NSASGDVADIKEGALYIVCAPSQG---------FDIYVNGYFRVYFKSVGNQ-
CpCDV [AM850136]    QIKSCNKFFKRLGVSTEWK-NSSTGDVGDIKEGALYIVGAPSQK-------SDVYVN--GYFRVYFKSVGNQ-
TbYDV [M81103]      QWRHVTKFFKRLGVSTEWK-NSSTGDVADIKEGALYLVCAPGGG---------ATVRVGGRFRMYFKSVGNQ-
CSMV [M20021]       PV--LNKFAKQLGVRTEWK-NAEGGDFGDIKSGALYLVMAPANG---------AVFVARGNVRVYFKSVGNQ-
DDSMV [HM122238]    PV--LNKFAKQLGVRTEWK-NTAGGDFGDIKSGALYLVLAPANG---------LTFVARGNIRMYFKSVGNQ-
PSMV [JF905486]     NLVSVTRFAKGLGVRTEWK-DTTTADASDIKGGALYLVVAPANG---------LVFTARGVIKVYFKSVGNQ-
BCSMV [HQ113104]    NLCTINRFAKGLGVRTEWK-DTVSADASDIKGGALYIVLAPANG---------LVFTARGVIKVYFKSVGNQ-
MRSV [JQ624880]     THLYFHKFAKGLGVRTEWK-NDTAGSVGNIKKGALYIGIAPGNG---------VEFNVFGKTRLYFKSIGNQ-
MSV [AF329881]      RNIYFHKFTSGLGVRTQWK-NVTDGGVGAIQRGALYMVIAPGNG---------LTFTAHGQTRLYFKSVGNQ-
SSEV [AF239159]     RAIYFHKFFTGLGVKTEWK-NLTDGGVGAIKKGALYLVIAPGNG---------LEFTCHGQARLYFKSVGNQ-
SSRV [AF072672]     KNIYFHKFVTGLGVKTEWK-NTTGGEVGDIKKGALYIVIAPGNG---------LDFTVHGNARLYFKSVGNQ-
SSV [M82918]        KNIYFHKFVTGLGVKTEWK-NTTGGEVGDIKKGALYIVIAPGNG---------LDFTVHGNARLYFKSVGNQ-
ESV [EU244915]      KNIYFHKFEAGLGVKTEWK-NTTGGDVGDIKKGALYIVIAPGNG---------LEFTVHGNARLYFKSVGNQ-
DSV [M23022]        RSIYFHKFATGLGVKTEWK-NTTDGGVGSIKKGALYFVIAPGSG---------IDFTLFGTCRMYFKSVGNQ-
PanSV [L39638]      KDIYFHKFCTGLGVKTEWK-NVTDGKVGAIKKGALYIVIAPGNG---------LEFTVHGQCRLYFKSVGNQ-
SacSV [GQ273988]    RSIYFHKFATGLGVKTEWK-NLADGGVGSIKKGALYIVIAPGNG---------LEFTAHGNARLYFKSVGNQ-
USV [EU445697]      RSIYFHKFVTGLGVKTEWK-NVADGGVGAIKKGALYLVIAPGNG---------LTFTAHGIARLYFKSVGNQ-
```

Mastrevirus

```
GRLaV-WA[JQ000000]  AA--FNIFQRR-RLVVAFKNDVSGGGRNDVERNRIYLSAASASG-----HTFRLYLNGIVN---FYNGVIFQ-
GCFaV-NY [JQ901105] AA--FNIFQRR-RLVVAFKNDVSGGGRNDVERNRIYLSAASASG-----HTFRLYLNGIVN---FYNGVIFQ-
```

Graingemvirus

```
SPLCV [AF104036]    ----IRKFFKGLYNHVTYN-HKEEAKYENQLENALMLYSASSHA------SNPVYQTL--RCRAYFYDSHNN-
CGMV [AF029217]     ----VKRFFRNINHRVVYN-QQEGAEYKNHHENALMLYMACSHA------SNPVYATI--KVRIYFYDSITN-
HYVV [AB236325]     ----VIKRFWKLNHHVTYN-HQEQAKYENHTENALLLYMACTHA------SNPVYATL--KIRVYFYDSVQN-
TLCSV [AY044137]    ----VIRRFYKIYNHIVYN-HQEQGKYENHTENALLLYMACTHA------SNPVYATL--KIRVYFYDSISN-
DoYMV [AM157413]    ----VIKRFWRVNNYVVHN-HQEAAKYENHTENALLLYMACTHA------SNPVYATL--KIXIYFYDSISN-
TLCYV [AJ512761]    ----IIRKFYRVNNYVVYN-HQEAGKYENHTENALLLYMACTHA------SNPVYATL--KVRSYFYDSVTN-
TGMV [K02029]       ----LVRRFWKVNNNVVYN-HQEAGKYENHTENALLLYMACTHA------SNPVYATL--KIRIYFYDSITN-
BGYMV [D00201]      ----LVRRFWKVNNHVVYN-HQEAGKYENHTENALLLYMACTHA------SNPVYATL--KIRIYFYDSITN-
TSLCV [AF130415]    ----IVKRFWRVNNHVVYN-HQEAGKYENHTENALLLYMACTHA------SNPVYATL--KIRIYFYDSVLN-
CoGMV [EU636712]    ----LVRRFWKVNNHVVYN-HQEAAKYDNHTENALLLYMACTHA------SNPVYATL--KIRIYFYDSILN-
MaMPRV [AY044133]   ----LVKRFWKVNNHVVYN-HQEAAKYENHTENALLLYMACTHA------SNPVYATL--KIRIYFYDSIMN-
TLCNDV [U15015]     ----LVRKFVRVNNYVVYN-QQEAGKYENHTENALMLYMACTHA------SNPVYATL--KIRIYFYDSATN-
CLCRV [AM501481]    ----LVKKFVRVNNYVVYN-QQEAGKYENHTENALMLYMACTHA------SNPVYATL--KIRIYFYDSVTN-
TLCJV [AB100304]    ----LVKRFFRVNNHVVYN-HQEAGKYENHTENALLLYMACTHA------SNPVYATL--KIRIYFYDSVQN-
CIGMV [DQ641692]    ----LVRRFIRVNNYVVYN-HQEAAKYENHTENALLLYMACTHA------SNPVYATL--KIRIYFYDSVTN-
TYLCCV [AF311734]   ----LVKRFIRLNTHVVYN-HQEQAKYENHTENALLLYMACTHA------SNPVYATL--KVRIYFYDSQMN-
ACMV [J02057]       ----LVKRFYRLNHHVTYN-HQEAGKYENHTENALLLYMACTHA------SNPVYATL--KIRIYFYDSIGN-
TYLCSV [X61153]     ----LLKRFFKINTHVVYN-HQEQAKYENHTENALLLYMACTHA------SNPVYATL--KIRIYFYDAVTN-
```

Begomovirus
