# Supplementary material for: A Leafhopper-Transmissible DNA Virus with Novel Evolutionary Lineage in the Family Geminiviridae Implicated in Grapevine Redleaf Disease by Next-Generation Sequencing
Source: PLoS One. 2013 Jun 5;8(6):e64194. doi: 10.1371/journal.pone.0064194 (PMC3673993; doi:10.1371/journal.pone.0064194)
Supplement: Table S1 — Classification of sequence reads. Classification and abundance of high-throughput sequence reads obtained from ribo-depleted and double depleted cDNA libraries derived from symptomatic and non-symptomatic grapevine leaves, when mapped to virus/viroid database. (PDF) [file pone.0064194.s004.pdf]

Table S1a. Classification of sequence reads. Classification and abundance of high-throughput sequence reads obtained from ribo-depleted and dual-depleted cDNA libraries derived from symptomatic and non-symptomatic grapevine leaves, when mapped to virus/viroid database.

**Category of sequence reads**	**Reads from ribo-depleted cDNA library**		**Reads from dual-depleted cDNA library**	
	**Symptoms**	**No Symptoms**	**Symptoms**	**No Symptoms**
**Total reads**	34,504,862	76,668,556	1,865,157	3,345,220
**Total hits to virus/viroid database**	53,988	16,268	3,232	2,539
**Unmapped reads**	34,465,910	76,652,288	1,862,331	3,342,861

Table S1b. Sequence reads summary for host subtraction and assembly approach.

**Category of sequence reads**	**Reads from ribo-depleted cDNA library**		**Reads from double depleted cDNA library**	
	**Symptoms**	**No Symptoms**	**Symptoms**	**No Symptoms**
**Total reads**	34,504,862	76,668,556	1,865,157	3,345,220
**Reads mapped to *Vitis* genome**	11,938,839	34,459,395	597,216	1,350,283
**Reads not mapped to *Vitis* genome (used in assembly)**	22,566,023	42,209,161	1,267,941	1,994,937
**# of Scaffolds assembled from non-*Vitis* reads**	20,066		436	
