# Supplementary figures and images for: Correction: A Leafhopper-Transmissible DNA Virus with Novel Evolutionary Lineage in the Family Geminiviridae Implicated in Grapevine Redleaf Disease by Next-Generation Sequencing
Source: PLoS One. 2016 Jan 15;11(1):e0147510. doi: 10.1371/journal.pone.0147510 (PMC4714755; doi:10.1371/journal.pone.0147510)

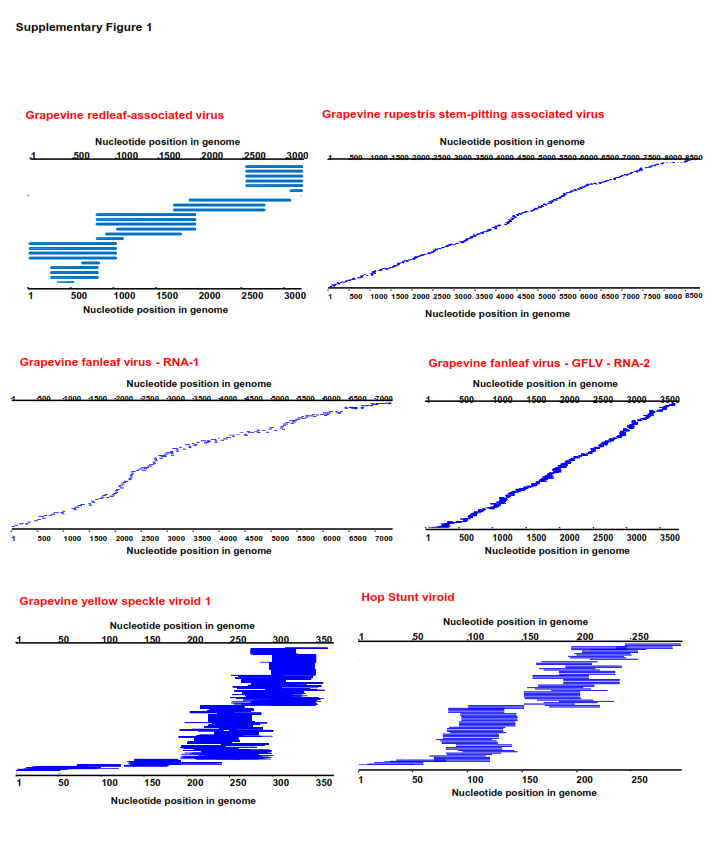

Supplement: S1 Fig — Nucleotide numbers of each virus and viroid genome is indicated at the top and bottom. Each bar represents the location of individual contigs aligning with the genome. (TIF) [file pone.0147510.s001.tif]
